# Supplementary material for: Socioeconomic and lifestyle factors associated with hearing loss in older adults: a cross-sectional study of the English Longitudinal Study of Ageing (ELSA)
Source: BMJ Open. 2019 Sep 17;9(9):e031030. doi: 10.1136/bmjopen-2019-031030 (PMC6756470; doi:10.1136/bmjopen-2019-031030)
Supplement: Author's manuscript [file bmjopen-2019-031030.draft_revisions.pdf]

BMJ Open is committed to open peer review. As part of this commitment we make the peer review history of every article we publish publicly available.

When an article is published we post the peer reviewers' comments and the authors' responses online. We also post the versions of the paper that were used during peer review. These are the versions that the peer review comments apply to.

The versions of the paper that follow are the versions that were submitted during the peer review process. They are not the versions of record or the final published versions. They should not be cited or distributed as the published version of this manuscript.

BMJ Open is an open access journal and the full, final, typeset and author-corrected version of record of the manuscript is available on our site with no access controls, subscription charges or pay-per-view fees (http://bmjopen.bmj.com).

If you have any questions on BMJ Open's open peer review process please email info.bmjopen@bmj.com

BMJ Open

Socioeconomic and Lifestyle Factors Associated with Hearing Loss in Older Adults: A Cross-sectional Study of the English Longitudinal Study of Ageing (ELSA)

Journal:	*BMJ Open*
Manuscript ID	bmjopen-2019-031030
Article Type:	Research
Date Submitted by the Author:	13-Apr-2019

Complete List of Authors:	Tsimpida, Dialechti; University of Manchester, Centre for Primary Care, Division of Population Health, Health Services Research & Primary Care, School of Health Sciences, Faculty of Biology, Medicine and Health<br>Kontopantelis, Evangelos; University of Manchester, Division of Informatics, Imaging & Data Sciences, School of Health Sciences, Faculty of Biology, Medicine and Health, The University of Manchester<br>Ashcroft, Darren; University of Manchester, Centre for Pharmacoepidemiology and Drug Safety, Division of Pharmacy and Optometry, School of Health Sciences, Faculty of Biology, Medicine and Health, The University of Manchester<br>Panagioti, Maria; University of Manchester, Centre for Primary Care, Division of Population Health, Health Services Research & Primary Care, School of Health Sciences, Faculty of Biology, Medicine and Health

Keywords:	PUBLIC HEALTH, SOCIAL MEDICINE, PREVENTIVE MEDICINE

SCHOLARONE™
Manuscripts

TITLE PAGE

Socioeconomic and Lifestyle Factors Associated with Hearing Loss in Older Adults: A Cross-sectional Study of the English Longitudinal Study of Ageing (ELSA)

Dialechti Tsimpida,*
NIHR Doctoral Research Fellow, Centre for Primary Care, Division of Population Health, Health Services Research & Primary Care, School of Health Sciences, Faculty of Biology, Medicine and Health, The University of Manchester, Manchester, UK
** Corresponding author: Centre for Primary Care, 5th floor Williamson Building, The University of Manchester, Oxford Road, M139PL, Manchester, UK*
Tel.: +44 161 275 7006
E-mail address: dialechti.tsimpida@manchester.ac.uk

Professor Evangelos Kontopantelis,
Division of Informatics, Imaging & Data Sciences, School of Health Sciences, Faculty of Biology, Medicine and Health, The University of Manchester, Manchester, UK
E-mail address: e.kontopantelis@manchester.ac.uk

Professor Darren Ashcroft,
Division of Pharmacy and Optometry, School of Health Sciences, Faculty of Biology, Medicine and Health, The University of Manchester, Manchester, UK
E-mail address: Darren.Ashcroft@manchester.ac.uk

Dr Maria Panagioti,
Senior Research Fellow, Centre for Primary Care, Division of Population Health, Health Services Research & Primary Care, School of Health Sciences, Faculty of Biology, Medicine and Health, The University of Manchester, Manchester, UK
E-mail address: maria.panagioti@manchester.ac.uk

Keywords: Hearing loss; socioeconomic position; hearing health inequalities; healthy ageing; social epidemiology

Word count for the abstract; **299**

Manuscript word count; **3.382**

Number of references; **34**

For peer review only - http://bmjopen.bmj.com/site/about/guidelines.xhtml

ABSTRACT

Objectives: Aims were (a) to examine whether socioeconomic position (SEP) is associated with hearing loss (HL) among older adults in England and (b) whether major modifiable lifestyle factors (such as high body mass index, physical inactivity, and excessive smoking and alcohol consumption) are associated with HL after controlling for the effects of non-modifiable demographic factors and SEP.

Setting: We used data from the wave 7 of the English Longitudinal Study of Aging (ELSA), which is a longitudinal household survey dataset of a representative sample of people aged 50 and older in England.

Participants: The final analytical sample was n=8,529 participants, aged 50-89, who gave their consent to have their hearing acuity objectively measured by a screening audiometry device and did not have any ear infection.

Primary and secondary outcome measures: HL defined as >35dB at 3.0 kHz, in the better-hearing ear. Those with HL were further subdivided into two categories depending on the number of tones heard at 3.0 kHz.

Results: HL was identified in 32.1% of men and 22.3% of women aged 50-89. Those in a lower SEP were up to two times more likely to have HL; the adjusted odds of HL were higher for those with no qualifications versus those with a degree/higher education, those in routine/manual occupations versus those in managerial/professional occupations, and those in the lowest versus the highest income and wealth quintiles. All regression models showed that socioeconomic and the modifiable lifestyle factors were strongly associated with HL after controlling for age and gender.

Conclusions: Our findings show that socioeconomic and lifestyle factors are associated with HL among older adults as strongly as core demographic risk factors, such as age and gender. Socioeconomic inequalities and modifiable lifestyle behaviours need to be targeted by health policy strategies, to improve the wellbeing of older populations.

For peer review only - http://bmjopen.bmj.com/site/about/guidelines.xhtml

Strengths and limitations of this study

- The first study that focuses on modifiable lifestyle factors (such as high body mass index, physical inactivity, and excessive smoking and alcohol consumption) associated with HL among older adults in the UK.
- Examines the effects of four different SEP indicators to HL (education, occupation, income, wealth), instead of a proxy measures to reflect one's total SEP, capturing therefore most of the variation in socioeconomic stratification, to the objectively measured HL in older adults.
- The analyses were based on a representative cohort of 8,529 participants contained in ELSA, which is a rich resource of information on the dynamics of health, social, wellbeing and economic circumstances in the English population aged 50 and older.
- The ELSA dataset did not contain information concerning the occupational and social noise exposure, which has a damaging effect in hearing, but we examined the association of manual occupations with HL and its attenuation by modifiable determinants including smoking habit, which is of a higher prevalence among those that work in routine and manual occupations in England.
- All the analysed factors explained less than one third of the variance for the occurrence of HL suggesting that there are additional major factors associated with HL in older adults which have not been included in our analyses.

For peer review only

For peer review only - http://bmjopen.bmj.com/site/about/guidelines.xhtml

INTRODUCTION

Hearing loss (HL) is a major global health challenge and the most prevalent sensory disorder. Approximately 15% of the global adult population has some degree of HL (of at least ≥25 dB HL in the better ear)[1] and almost 7% has disabling HL (defined as a hearing threshold ≥40 dB HL in the better ear)[2]. HL has been associated with negative physical, social, cognitive, economic and emotional consequences and is the fourth leading contributor to years lived with disability worldwide[2].

Previous studies have reported that HL increases with age[3], exposure to high occupational and social noise[4] and occurs more commonly among men[3]. There is growing evidence that there are a number of modifiable risk factors resulting in the aetiology of HL[5,6] and, if eliminated, might prevent more than the half cases of HL[2]. It can thus be suggested that there is a high potential for reducing the burden of HL, if we understand the modifiable factors and the mechanisms that lead to hearing health inequalities, which -following the glossary for health inequalities[7]- could be defined as the *avoidable differences in people's hearing health across different social and/or population groups*. Prior research has established health disparities in a wide range of health conditions according to socioeconomic position (SEP)[8]. However, hearing health inequalities is an emerging research area and the existing evidence on the relationship of HL with SEP and modifiable lifestyle factors is limited. There is a major public health need to assess whether HL is associated with SEP and lifestyle factors because this understanding could inform recommendations for HL preventative strategies such as wider implementation of interventions to promote 'healthier lifestyles' and governmental policies for socioeconomic equity among older people in the community.

The aims of this study were (a) to examine whether SEP is associated with HL among older adults in England and (b) whether major modifiable lifestyle factors are associated with HL after controlling for the effects of non-modifiable demographic factors and SEP. This study is the first that examines the effects of four different SEP indicators (education, occupation, income, wealth), encompassing thus aspects of the life-course socioeconomic stratification[9], to the objectively measured HL in older adults. In addition, is the first study that explores how major

For peer review only - http://bmjopen.bmj.com/site/about/guidelines.xhtml

lifestyle factors for general health outcomes in the English adult population (such as smoking, high BMI, insufficient physical activity and excessive alcohol consumption)[10,11] account for the variance in HL.

METHODS

Study population

The present study used data from the English Longitudinal Study of Aging (ELSA). The ELSA is a longitudinal household survey dataset of a representative sample of people aged 50 and older in England. It is designed as a large-scale prospective cohort study, with repeat measures of core variables over numerous waves, in order to explore trajectories on the health, social, wellbeing and economic circumstances. The current sample contains data from up to eight waves of data collection covering a period of 15 years, with an ongoing two-year follow-up longitudinal design[12].

Objective hearing health data was available only in wave 7, where information was collected from 9,666 participants, between June 2014 and May 2015. For the purposes of this study, the final analytical sample was n=8,529 participants, aged 50-89, who gave their consent to have their hearing acuity measured by a screening audiometry device and did not have any ear infection.

Patient and Public Involvement

Patients were not involved in the conduct of the study.

Hearing test

A handheld audiometric screening device (HearCheck)[13] was used for the objective measurement of hearing acuity. This is a hearing screening test by Siemens, that tests for

For peer review only - http://bmjopen.bmj.com/site/about/guidelines.xhtml

audibility of pure tone beeps, according to the number of tones that the respondent can hear for each sequence (at 1.0 kHz and 3.0 k Hz), per each ear. The functional test sequence begins with a series of three sounds, that have decreasing volume at 1.0 kHz (55 dBHL, 34 dBHL, 20 dBHL) and afterwards another three sounds with decreasing volume at 3.0 kHz (75 dBHL, 55 dBHL, 35 dBHL). Prerequisites for the test were the device to make proper contact with the ear that is tested, hearing aid(s), glasses, earrings and hair bands to be removed to prevent from getting in the way of the hearing device and the room to be as quiet as possible. Participants indicated when they hear the sound by raising their finger. The total number of tones that the participants indicated they could hear in the sequence of sounds at 1.0 kHz and 3.0. KHz, per each ear, was recorded and the total tones heard in the better ear used for the categorization of those with HL.

Outcomes

Hearing loss

HL was defined as >35dB at 3.0 kHz, in the better-hearing ear. This is the level where intervention for HL has been shown as definite beneficial[14] and has also been previously used for the categorization of those with HL[6]. Those with HL were further subdivided into two categories depending on the number of tones heard at 3.0 kHz. Thus, we further explored potential differences in the association between SEP indicators and HL, according to the severity of HL, as measured by HearCheck. The categorization of those with HL was as following:

(a) "*Moderate HL*": tones heard at 75 dBHL and 55 dBHL but not at 35 dBHL (the first 2 of the three tones at 3.0 kHz heard),

(b) "*Moderately severe or severe HL*": tone heard or not at 75 dBHL and tones not heard at 55 dBHL and 35 dBHL (0 or 1 of the three tones at 3.0 kHz heard). The category of "normal hearing", along with the above two categories of HL, constituted the ordinal variable "hearing acuity" (in better ear).

For peer review only - http://bmjopen.bmj.com/site/about/guidelines.xhtml

Indicators of socioeconomic position

Education, occupation, income and wealth were the four selected indicators of SEP. We considered five categories of the highest educational attainment: degree/higher education; A level; O levels CSE; foreign/other; no qualifications. Tertiles of self-reported occupation were based on the National Statistics socio-economic classification (NS-SEC): managerial and professional; intermediate; routine and manual occupations). This was based on the household reference person with the highest income. Thus, it was possible that the household reference person was a woman. The relative financial position of the participants was captured by quintiles of the net household income (first quintile lowest; fifth quintile highest) that is summed across household members. In order to avoid the information bias that is related to the retirement status, we used quintiles of the total non-pension wealth that is reported at the household level (first quintile lowest; fifth quintile highest), which represents the sum of net financial wealth, net physical wealth and net housing wealth.

Covariates

Age, marital status, retirement status and non-medical determinants of health (body mass index, physical activity, tobacco and alcohol consumption) were assessed as covariates in the association between SEP indicators and HL[5].

Marital status was dichotomised into those that are currently married or not. Those who are currently married included the categories a) married, first and only marriage, b) in a registered Civil Partnership, c) remarried, in a second or later marriage. Those that categorised as not currently married included the categories a) single, that is never married and never registered in a marriage, b) separated, but still legally married, c) divorced, d) widowed.

Retirement status was dichotomised into those who were retired or not, according to the self-reported employment status.

For peer review only - http://bmjopen.bmj.com/site/about/guidelines.xhtml

Body Mass Index (BMI) measurements were grouped in four categories, according to WHO definitions[15]: (a) underweight: BMI under 18.5, (b) normal: BMI 18.5 or over but less than 25, (c) overweight: BMI 25 or over but less than 30, and (d) obese: BMI 30 or over.

Tobacco consumption of any type of nicotine products was recoded into three categories: those that were current smokers, those that were former smokers and those that never smoked. Both current and former smokers answered the question of 'number of cigarettes smoked per day', to explore whether they were occasional or regular smokers.

Alcohol consumption was recorded using several continuous variables such as the number of days of alcohol consumption in the last seven days and the number of (a) measures of spirit, (b) glasses of wine and (c) pints of beer that the respondents had consumed during this period. We constructed a continuous variable to represent the sum of units of alcohol that the participants consumed in the last seven days, according to the Chief Medical Officer's Drinking Guidelines[16], that counts as 1 unit each measure of spirit and as 2 units each glass of wine of pint of beer. The constructed variable of units of alcohol during the last seven days was further dichotomised into those that consumed more than 14 units of alcohol the last seven days or not, in a separate variable.

Levels of physical activity were described by three ordinal variables that examined the frequency that the respondents do rigorous, moderate or mild sports or activities, with possible answers (a) more than once a week, (b) once a week, (c) one to three times a month and (d) hardly ever, or never.

Statistical analysis

Categorical variables are presented as absolute (n) and relative (%) frequencies, while continuous variables are presented using their mean and standard deviation. The Kolmogorov-Smirnov test and normal plots were used to test the normality of the quantitative variable distributions. There were no missing values in the hearing data of the final analytical sample (n=8,529). Due to low proportion of missingness (<5%) in any of the other variables, records with missing data were dropped from analysis.

For peer review only - http://bmjopen.bmj.com/site/about/guidelines.xhtml

We fitted multiple logistic regression models to evaluate the odds of HL at various socioeconomic strata, controlling for gender, age and non-medical determinants of health (BMI, physical activity, tobacco and alcohol consumption). Additionally, we fitted four separate stepwise logistic regression models, to examine the association of HL with non-modifiable (age, gender: Step 1), partly modifiable (education, occupation, income, wealth: Step 2, respectively), and fully modifiable lifestyle risk factors (body mass index, physical activity, and smoking and alcohol consumption: Step 3). The variance inflation factor (VIF) was used as an indicator of multicollinearity and the Hosmer-Lemeshow test was used as a post estimation tool, which quantified the goodness-of-fit of the models. For all models, odds ratios, 95% confidence intervals, unadjusted and adjusted coefficients' beta values, pseudo R^2 and mean VIFs are presented. The two-tailed significance level was set ≤0.05. All data were analysed using Stata version 14 (StataCorp, 2015)[17].

RESULTS

Socio-demographic characteristics

Table 1 shows the distribution of socio-demographic characteristics of the sample (n=8,529, aged 50-89). Overall, 26.6% (2,266/8,529) of adults aged 50-89 had HL >35dB at 3.0 kHz. The percentages were 32.1% (1,198/3,728, 95%CI 0.306 to 0.337) for men and 22.3% (1,068/4,801, 95%CI 0.211 to 0.234) for women, respectively. Men were twice as likely to have moderately severe or severe HL compared to women. Also the percentage of HL in age band 75-89 were fivefold larger than those of age band 50-64, with one out of every two adults aged 75-89 having HL >35dB at 3.0kHz.

Table 1

For peer review only - http://bmjopen.bmj.com/site/about/guidelines.xhtml

Lifestyle factors

Lifestyle factors of the participants are presented in Table 2. Over half of the participants were current or former smokers. In addition, patterns of high levels of alcohol consumption among all participants were revealed, with average consumption of more than 14 units of alcohol in the last seven days for two out of three participants (5,223/8,528, 95%CI 0.602 to 0.613). Nearly one out of every three of the excessive drinkers (1,457/5.223, 95%CI 0.267 to 0.291) had HL >35dB at 3.0 kHz.

Three out of four of those with HL >35dB at 3.0 kHz were overweight or obese. Furthermore, those with HL >35dB at 3.0 kHz were twice as likely to hardly ever or never engage in moderate or mild sports activities compared to hearing participants.

Table 2

Hearing Loss

Table 3 and Figure 1 show the results of multiple logistic regression analysis with HL >35dB at 3.0 kHz as the dependent variable and SEP indicators as the independent variables, per each gender. The adjusted odds of HL were higher for those with no qualifications versus those with a degree/higher education (men: OR 1.87, 95% CI 1.47-2.38, women: OR 1.53, 95% CI 1.21-1.95), those in routine/manual occupations versus those in managerial/professional occupations (men: OR 1.92, 95% CI 1.43-2.63, women: OR 1.25, 95% CI 1.03-1.54), and those in the lowest versus the highest income and wealth quintiles (men: OR 1.62, 95% CI 1.08-2.44, women: OR 1.36, 95% CI 0.85-2.16 and men: OR 1.72, 95% CI 1.26-2.35, women: OR 1.88, 95% CI 1.37-2.58, respectively).

Table 3 & Figure 1

For peer review only - http://bmjopen.bmj.com/site/about/guidelines.xhtml

Table 4 shows the summary of stepwise logistic regression analysis for variables predicting HL >35dB at 3.0 kHz. All regression models were statistically significant. Age and gender only explained about 15% of the variance in the likelihood of HL. The addition of SEP and lifestyle factors in the regression models explained another 10 to 15% of the variance. The total variance explained in the overall models containing demographic factors, SEP and lifestyle factors ranged between 25 and 27%. This finding suggests that SEP and lifestyle factors have an equal contribution to HL as age and gender.

Table 4

DISCUSSION

Summary of main findings

In this study we examined whether SEP and modifiable lifestyle factors are associated with HL among older adults in the UK. We found that variation in education, occupation, income and wealth, which are important determinants of health inequality, are associated with HL. SEP was strongly associated with the likelihood of HL in older adults, with the higher levels of education, income and wealth being less likely to be associated with HL, and the manual occupations increased the likelihood of HL. We also found that several modifiable lifestyle factors (such as high body mass index, physical inactivity, and excessive smoking and alcohol consumption) are associated with the likelihood of HL as strongly as well-established demographic factors such as age and gender HL. These findings suggest that a large proportion of HL burden is potentially preventable and support the proposition of Scholes et al.[6] that there is serious potential to reduce the prevalence and impacts of HL by understanding the impact of socioeconomic inequality in hearing health. Thus the incidence and severity of HL in the UK could be significantly reduced by governmental policies to mitigate socioeconomic disparities and public health interventions to promote healthier lifestyles in middle-aged and older adults

For peer review only - http://bmjopen.bmj.com/site/about/guidelines.xhtml

For peer review only

in the UK. The occurrence of objective hearing data eliminated the different types of bias that occur in self-reporting hearing difficulties[18], strengthening the accuracy of findings.

Strengths and limitations

The main strength of our study was that is the first to focus on modifiable lifestyle factors associated with HL among older adults in the UK and to examine the effects of four different SEP indicators to HL, instead of a proxy measures to reflect one's total SEP[14], capturing therefore most of the variation in socioeconomic stratification[9]. Another strength is that the analyses were based on a representative cohort of 8,529 participants contained in ELSA, which is a rich resource of information on the dynamics of health, social, wellbeing and economic circumstances in the English population aged 50 and older[12].

However there are also important limitations. First, no causal or temporal relationships can be established between lifestyle factors and HL in this cross-sectional study. Unhealthy lifestyle behaviours could lead to HL in older people but is also possible that older people adopt less healthy lifestyles after HL. Second, all the analysed factors explained less than one third of the variance for the occurrence of HL suggesting that there are additional major factors associated with HL in older adults which have not been included in our analyses. Longitudinal analyses using a broader range of physical health, mental health and social care variables are highly recommended to obtain a comprehensive understanding of modifiable factors which contribute to HL among older adults in the UK. Third, the ELSA dataset did not contain information concerning the occupational and social noise exposure, which has a damaging effect in hearing[4]. We therefore were not able to examine the association of noise exposure with smoking in the relationship of SEP with HL, as in a previous study which found that the smoking habit in workers exposed to occupational noise greatly influenced HL[19]. However, we examined the association of manual occupations with HL and its attenuation by modifiable determinants including smoking habit, which is of a higher prevalence among those that work in routine and manual occupations in England[10].

For peer review only - http://bmjopen.bmj.com/site/about/guidelines.xhtml

Research and policy implications

A number of previous studies have reported that the odds of HL in older adults were significantly increased for those with lower educational attainment[6,20,21,22], and those in manual versus non-manual occupations[23,24,25,26]. Besides, income has also been reported as a correlate of HL, with the prevalence of untreated HL being higher among low-income older adults in the United States[27]. In our study, those in the lowest quintile of net household income had disproportionally higher percentages of moderate HL compared to moderately severe or severe HL, but this pattern was not revealed in the quintiles of wealth, as expected. This may reveal a possible delay in diagnosis of hearing problems among those in lower SEP due to financial barriers in access to health services[28], which needs further exploration, as HL is highly undiagnosed and untreated among older adults in England[18].

International studies have also shown that smoke consumption, high body mass and high fat and high calorie food consumption can have an extensive impact on hearing[29,30,31,32]. On the other hand, a higher level of physical activity is related inversely with risk of HL[31]. In our study, two out of three of the participants were drinking above the Chief Medical Officer's Drinking Guidelines[16], which is not to drink more than 14 units of alcohol a week. We considered therefore that excessive alcohol consumption may play an important role in the association between SEP and HL among the English population and thus we included this variable in the regression models, which has not been previously examined in the literature. The higher prevalence of HL among men aged 50 and above compared to women has also been reported in other studies[3,6]. However, we noticed that the rate of deterioration of hearing acuity as age increases was similar between each age band and nearly to 60% in both genders, as Figure 2 shows. The difference in prevalence begins at the age band "50-64", where men were twice as likely to have HL. Thus, the differences in modifiable lifestyle factors that were revealed in the stepwise regression models may finally explain why the male sex is not a consistent risk factor in studies[26], leading to the exploration of modifiable determinants that are common in both genders[5].

For peer review only - http://bmjopen.bmj.com/site/about/guidelines.xhtml

In terms of policy, providing evidence concerning the critical variables associated with HL is an important step in designing universal and targeted services and interventions for individuals that face hearing health inequalities, ensuring the wellbeing of older populations, and especially of those in the lowest SEP groups, were burden of HL fall highest. This is of major importance for the population in England, as the sensor diseases are the first leading cause of morbidity among adults 70 years and older and the second leading cause among adults 50-69 years 10. Our findings provide support to the view that HL is a non-communicable disease[33] which can be prevented or ameliorated by governmental policies to mitigate socioeconomic disparities and public health interventions to promote healthier lifestyles in middle-aged and older adults in the UK.

Conclusion

The main finding of our study is that HL is strongly associated with socioeconomic factors and modifiable lifestyle behaviours. Our findings are supportive of a new conceptualisation of HL which argues that HL is not necessarily an inevitable accompaniment of ageing, but also a potential preventable lifestyle disease, paving the way for the term *lifestyle-related hearing loss*, where *lifestyle* refers to *social practices and ways of living adopted by individuals that reflect personal, group, and socio-economic identities*[34], instead of the non-inclusive term "*age-related hearing loss*". Future research in hearing health inequalities should investigate the role of the prolonged exposure to these modifiable lifestyle behaviours in the development of HL.

For peer review only - http://bmjopen.bmj.com/site/about/guidelines.xhtml

Contributors DT, EK, DA and MP were responsible for developing the design of the study. DT was responsible for conducting the analyses, interpreting the results and drafting the manuscript. DT, EK, DA and MP critically revised the manuscript. All authors have read and approved the final manuscript.

Non-author contributors DT would like to acknowledge the contribution of Dr Piers Dawes and Prof Neil Pendleton, who had provided assistance in obtaining funding and were the former supervisors over her NIHR Manchester Biomedical Research Centre PhD Studentship, but did not fulfil the criteria set out in the Authorship Guidelines of The University of Manchester to be listed as authors.

Funding This research was funded by the NIHR Manchester Biomedical Research Centre (BRC). The views expressed are those of the authors and not necessarily those of the BRC, the NIHR or the Department of Health.

Competing interests None declared

Patient consent Not required

Ethics approval Not required. The study used data from the English Longitudinal Study of Ageing (ELSA).

Provenance and peer review Non commissioned; externally peer reviewed

Data sharing statement The English Longitudinal Study of Ageing dataset is available via the UK Data Service (http://www.ukdataservice.ac.uk). Statistical code is available from the corresponding author at dialechti.tsimpida@manchester.ac.uk.

Open Access This is an Open Access article distributed in accordance with the Creative Commons Attribution Non Commercial (CC BY-NC 4.0) license, which permits others to distribute, remix, adapt, build upon this work non-commercially, and licence their derivative works on different terms, provided the original work is properly cited and the use is non-commercial. See: http://creativecommons.org/licenses/by-nc/4.0/

© Article author(s) (or their employer(s) unless otherwise stated in the text of the article) 2019. All rights reserved. No commercial use is permitted unless otherwise expressly granted.

For peer review only - http://bmjopen.bmj.com/site/about/guidelines.xhtml

Table 1
Participants socio-demographic characteristics (N=8,529, aged 50-89)

Variable	Hearing acuity % (N)			
	Normal Hearing	HL >35dB at 3.0kHz	Moderate HL	Moderately severe or severe HL
**Gender**				
Male	67.87 (2,530)	32.14 (1,198)	19.88 (741)	12.26 (457)
Female	77.76 (3,733)	22.25 (1,068)	15.77 (757)	6.48 (311)
**Age**[a]	64.28 (9.29)	69.70 (19.19)	69.99 (15.85)	69.13 (24.41)
**Age group**				
50-64	89.98 (3,135)	10.02 (349)	8.04 (280)	1.98 (69)
65-74	74.49 (2,108)	25.51 (722)	18.90 (535)	6.61 (187)
75-89	44.54 (868)	55.46 (1,081)	32.63 (636)	22.83 (445)
**Currently married**				
No	69.79 (1,908)	30.21 (826)	19.90 (544)	10.31 (282)
Yes	76.01 (4,202)	23.99 (1,609)	16.41 (907)	7.58 (701)
**Retirement status**				
Retired	65.54 (3,205)	34.46 (1,685)	22.74 (1,112)	11.72 (573)
Not retired	86.15 (2,905)	13.85 (467)	10.05 (339)	3.80 (128)
**Education**				
Degree/Higher Education	78.03 (1,996)	21.97 (562)	15.79 (404)	6.18 (158)
A level	81.31 (596)	18.69 (137)	13.64 (100)	5.05 (37)
O level/CSE grade	75.38 (1,448)	24.62 (473)	16.71 (321)	7.91 (152)
Foreign/Other	76.00 (798)	24.00 (252)	16.29 (171)	7.71 (81)
No qualifications	60.86 (1,090)	39.14 (701)	24.51 (439)	14.63 (262)
**Occupation based National Statistics Socio-economic Classification (NS-SEC)**				
Managerial and professional occupations	73.24 (1,158)	26.76 (423)	18.03 (285)	8.73 (138)
Intermediate occupations (non-manual)	76.37 (2,149)	23.63 (665)	16.95 (477)	6.68 (188)
Routine and manual occupations	65.11 (1,644)	34.89 (1,643)	22.02 (1,318)	12.87 (325)

(Continued)

For peer review only - http://bmjopen.bmj.com/site/about/guidelines.xhtml

Table 1 (Continued)
Distribution of SEP indicators, demographic characteristics and other covariates used in the analysis (n=8,529, aged 50-89)

Variable	Normal Hearing	Hearing acuity (%) HL >35dB at 3.0kHz	Moderate HL	Moderately severe or severe HL
**Net Household Income**				
First quintile (lowest)	67.44 (872)	32.56 (421)	20.26 (262)	12.30 (159)
Second quintile	66.23 (959)	33.77 (489)	22.72 (329)	11.05 (160)
Third quintile	69.54 (1,034)	30.46 (453)	19.97 (297)	10.49 (156)
Fourth quintile	75.87 (1,154)	24.13 (367)	17.42 (265)	6.71 (102)
Fifth quintile (highest)	82.07 (1,112)	17.94 (243)	13.14 (178)	4.80 (65)
**Net Financial Wealth**				
First quintile (lowest)	73.25 (794)	26.75 (290)	18.36 (199)	8.39 (91)
Second quintile	64.92 (879)	35.08 (475)	21.71 (294)	13.37 (181)
Third quintile	68.34 (1,006)	31.66 (466)	21.13 (311)	10.53 (155)
Fourth quintile	75.06 (1,204)	24.94 (400)	17.71 (284)	7.23 (116)
Fifth quintile (highest)	78.49 (1,248)	21.51 (342)	15.28 (243)	6.23 (99)

Values are expressed as n (%) unless otherwise is indicated.

[a]Mean (Standard deviation)

For peer review only

Table 2
Participants' lifestyle factors (N=8,529, aged 50-89)

Variable	Hearing acuity % (N) Normal Hearing	HL >35dB at 3.0kHz	Moderate HL	Moderately severe or severe HL
**Tobacco consumption (any type of nicotine products)**				
Current	76.81 (712)	23.19 (215)	14.99 (139)	8.20 (76)
Former	71.08 (2,996)	28.92 (1,219)	19.22 (810)	9.70 (409)
Number of cigarettes smoked per day [a]	12.79 (14)	12.79 (13)	12.69 (13)	11.90 (12)
Never	76.99 (2,403)	23.01 (718)	16.08 (502)	6.92 (216)
**Alcohol consumption (in the last 7 days)**				
Number of days of alcohol consumption [b]	3 (3)	3 (4)	3 (4)	3 (4)
Number of measures of spirit [a]	2.14 (2)	2.30 (3)	2.15 (3)	2.61 (3)
Number of glasses of wine [a]	4.30 (6)	3.62 (5)	3.88 (6)	3.08 (4)
Number of pints of beer [a]	2.11 (2)	2.34 (3)	2.33 (3)	2.35 (3)
Total units of alcohol in the last 7 days [a]	14.96 (18)	14.20 (19)	14.54 (21)	13.47 (17)
Consumption of more than 14 units	72.10 (3,766)	27.90 (1,457)	18.71 (977)	9.19 (480)
**BMI Classification**				
Underweight	67.35 (33)	32.66 (16)	16.33 (8)	16.33 (8)
Normal	76.95 (1,255)	23.05 (476)	14.65 (239)	8.40 (237)
Overweight	70.55 (1,869)	29.44 (780)	19.10 (506)	10.34 (274)
Obese	70.70 (1,390)	29.30 (576)	21.16 (416)	8.14 (160)
**Physical Activity**				
Frequency does rigorous sports or activities				
More than once a week	82.09 (1,407)	17.91 (307)	13.59 (233)	4.32 (74)
Once a week	80.57 (626)	19.43 (151)	14.80 (115)	4.63 (36)
One to three times a month	80.13 (617)	19.87 (153)	14.42 (111)	5.45 (42)
Hardly ever, or never	69.18 (3,459)	30.82 (1541)	19.84 (992)	10.98 (549)

(Continued)

For peer review only - http://bmjopen.bmj.com/site/about/guidelines.xhtml

Table 2 (Continued)

Participants' lifestyle factors (N=8,529, aged 50-89)

Variable	Normal Hearing	Hearing acuity (%)<br>HL >35dB at 3.0kHz	Moderate HL	Moderately severe or severe HL
**Physical Activity** (continued)				
Frequency does moderate sports or activities				
More than once a week	79.11 (4,180)	20.89 (1104)	14.76 (780)	6.13 (324)
Once a week	72.53 (771)	27.47 (292)	19.19 (204)	8.28 (88)
One to three times a month	68.05 (360)	31.94 (169)	20.79 (110)	11.15 (59)
Hardly ever, or never	57.65 (799)	27.28 (587)	25.76 (357)	16.59 (230)
Frequency does mild sports or activities				
More than once a week	76.38 (5,130)	23.62 (1,586)	16.43 (1,103)	7.19 (483)
Once a week	69.75 (498)	30.25 (216)	19.89 (142)	10.36 (74)
One to three times a month	64.65 (139)	35.35 (76)	22.33 (48)	13.02 (28)
Hardly ever, or never	55.59 (343)	44.41 (274)	25.61 (158)	18.80 (116)

Values are expressed as n (%) unless otherwise is indicated.

[a]Mean (Standard Error of Mean)

[b]Median (Range)

For peer review only

For peer review only - http://bmjopen.bmj.com/site/about/guidelines.xhtml

Table 3.
Multiple logistic regression analysis of N=8,529, aged 50-89 with **HL >35dB at 3.0kHz** as dependent variable and **SEP indicators** as independent variables

	**Unadjusted OR (95% CI)***		**Adjusted OR (95% CI)****	
	Men	Women	Men	Women
**Education**				
No qualifications	2.39 (1.96-2.90)	2.67 (2.20-3.24)	1.87 (1.47-2.38)	1.53 (1.21-1.95)
Foreign/Other	1.06 (0.83-1.36)	1.37 (1.07-1.74)	1.46 (1.09-1.94)	0.99 (0.74-1.32)
O level/CSE grade	1.56 (1.29-1.89)	1.00 (0.80-1.25)	1.42 (1.13-1.79)	0.94 (0.73-1.22)
A level	1.01 (0.77-1.32)	0.69 (0.50-0.97)	1.08 (0.78-1.51)	0.82 (0.56-1.21)
Degree/Higher Education (reference)				
**Occupation based National Statistics socio-economic classification (NS-SEC)**				
Routine and manual occupations	1.69 (1.39-2.08)	1.35 (1.15-1.59)	1.92 (1.43-2.63)	1.25 (1.03-1.54)
Intermediate occupations (non-manual)	1.47 (1.23-1.75)	1.54 (1.19-1.96)	1.61 (1.25-2.08)	1.35 (1.01-1.85)
Managerial and professional occupations (reference)				
**Net Household Income**				
First quintile (lowest)	1.94 (1.50-2.52)	3.04 (2.31-3.99)	1.62 (1.08-2.44)	1.36 (0.85-2.16)
Second quintile	2.12 (1.67-2.70)	3.00 (2.28-3.93)	1.31 (0.93-1.85)	1.40 (0.89-2.18)
Third quintile	1.98 (1.56-2.51)	2.31 (1.75-3.05)	1.40 (1.01-1.94)	1.08 (0.69-1.67)
Fourth quintile	1.38 (1.08-1.74)	1.65 (1.23-2.20)	1.09 (0.80-1.49)	1.08 (0.70-1.66)
Fifth quintile (highest) (reference)				
**Net Financial Wealth**				
First quintile (lowest)	1.11 (0.86-1.45)	1.79 (1.38-2.33)	1.72 (1.26-2.35)	1.88 (1.37-2.58)
Second quintile	1.92 (1.52-2.42)	2.39 (1.88-3.04)	1.66 (1.26-2.18)	1.33 (1.00-1.77)
Third quintile	1.63 (1.30-2.04)	1.95 (1.53-2.50)	1.45 (1.12-1.88)	1.41 (1.06-1.88)
Fourth quintile	1.06 (0.85-1.32)	1.48 (1.15-1.91)	0.96 (0.75-1.24)	1.26 (0.94-1.68)
Fifth quintile (highest) (reference)				

*Unadjusted odds ratio (OR) ** Odds Ratio adjusted for age, marital status, retirement status, body mass index, tobacco consumption, alcohol consumption and physical activity.

For peer review only - http://bmjopen.bmj.com/site/about/guidelines.xhtml
For peer review only

Table 4.
Summary of stepwise logistic regression coefficients for variables predicting **HL >35dB at 3.0kHz** (N=8,529, aged 50-89), according to different SEP indicators (education, occupation, income, wealth)

		Model A			Model B			Model C			Model D		
	**Step/Predictor**	**Step 1**	**Step 2a**	**Step 3**	**Step 1**	**Step 2b**	**Step 3**	**Step 1**	**Step 2c**	**Step 3**	**Step 1**	**Step 2d**	**Step 3**
**1**	**Non-modifiable**		(Education)			(Occupation)			(Income)			(Wealth)	
	Gender (female)	-.62***	-.59***	-.72***	-.62***	-.64***	-.68***	-.62***	-.69***	-.70***	-.62***	-.69***	-.62***
	Age (mean)	.12***	.11***	.10***	.12***	.13***	.11***	.12***	.11***	.11***	.12***	.11***	.12***
**2**	**Partly modifiable**												
2a	Education		-.15***	-.11***		-	-		-	-		-	-
2b	Occupation (manual)			-		.26***	.20***		-	-		-	-
2c	Net Household Income			-		-	-		-.14***	-.09***		-	-
2d	Net Financial Wealth			-		-	-		-	-		-.17***	-.11***
**3**	**Modifiable**												
	Smoking (current/former)			.10*			.09			.10*			.09**
	Alcohol consumption (> 14 units per week)			.24***			.19***			.17***			.18**
	Body mass index (<25)			-.05*			-.06			-.03			-.04
	Physical Activity (*rigorous* sports or activities, once or more/week)			-.14***			-.16***			-.12***			-.13***
	Physical Activity (*moderate* sports or activities, once or more/week)			-.24***			-.24***			-.24***			-.24***
	Physical Activity (*mild* sports or activities, once or more/week)			-.17***			-.15***			-.15***			-.14***
	Pseudo R^2	.15	.18	.28	.15	.19	.26	.17	.18	.29	.17	.18	.27
	Δ Pseudo R^2	-	.03	.10	-	.04	.07	-	.01	.11	-	.01	.09
	Mean VIF	-	-	1.16	-	-	1.20	-	-	1.24	-	-	1.15

*p < .05. **p < .01 ***p < .001

For peer review only - http://bmjopen.bmj.com/site/about/guidelines.xhtml

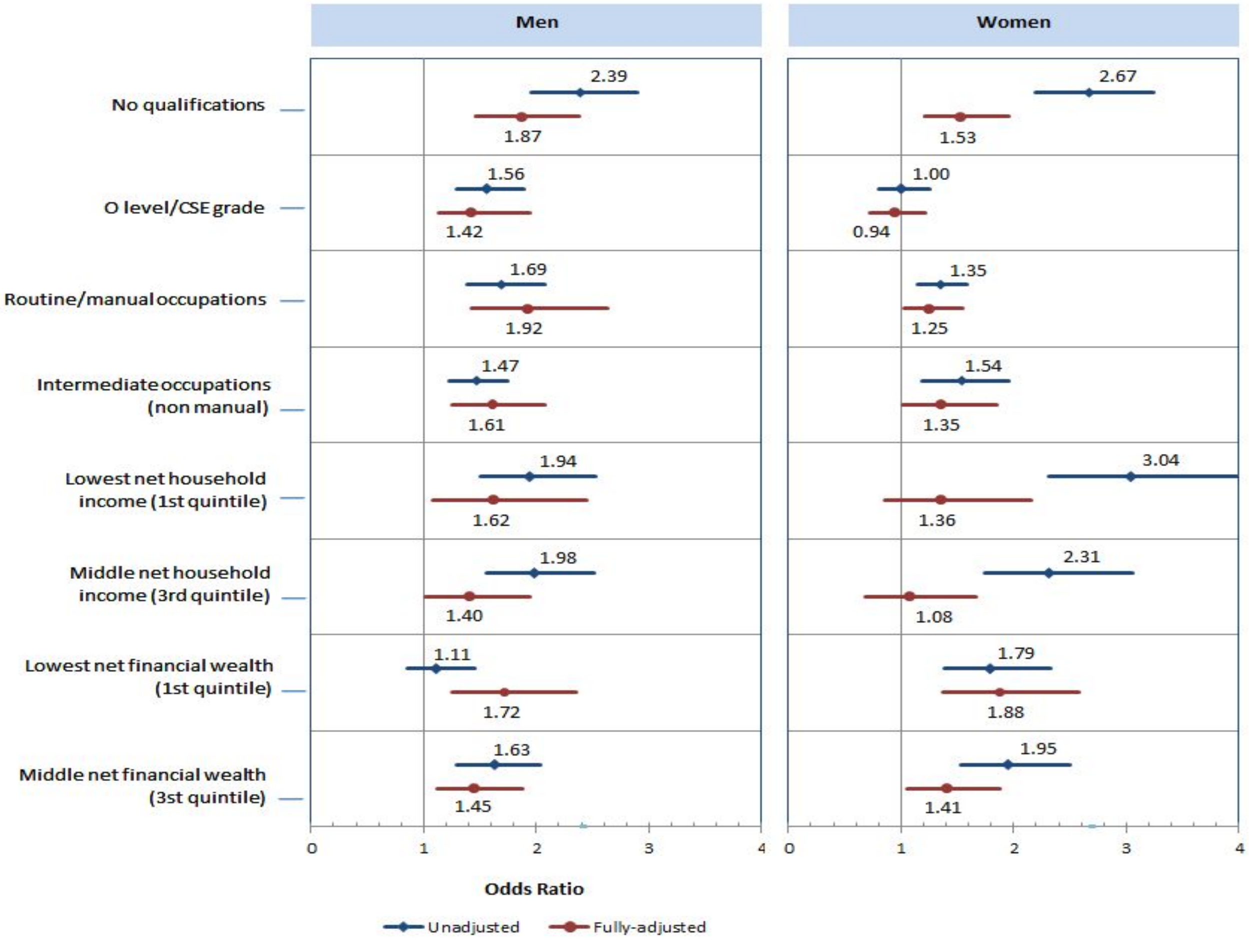

Figure 1 Associations between socioeconomic position and hearing loss in middle aged and older adults (N=8,529, aged 50-89). Indicators of SEP were categories of the highest educational attainment (degree/higher education as a reference), tertiles of self-reported occupation based on the National Statistics Socio-economic Classification (NS-SEC) (managerial and professional as reference), quintiles of the net household income (first quintile lowest; fifth quintile highest) and quintiles of the total non-pension wealth that is reported at the household level (first quintile lowest; fifth quintile highest). Lines represent OR (outcome=hearing loss) and its 95% CI. Model A (rhombus): unadjusted. Model B (circles): adjusted for age, marital status, retirement status, body mass index, tobacco consumption, alcohol consumption and physical activity.

For peer review only - http://bmjopen.bmj.com/site/about/guidelines.xhtml

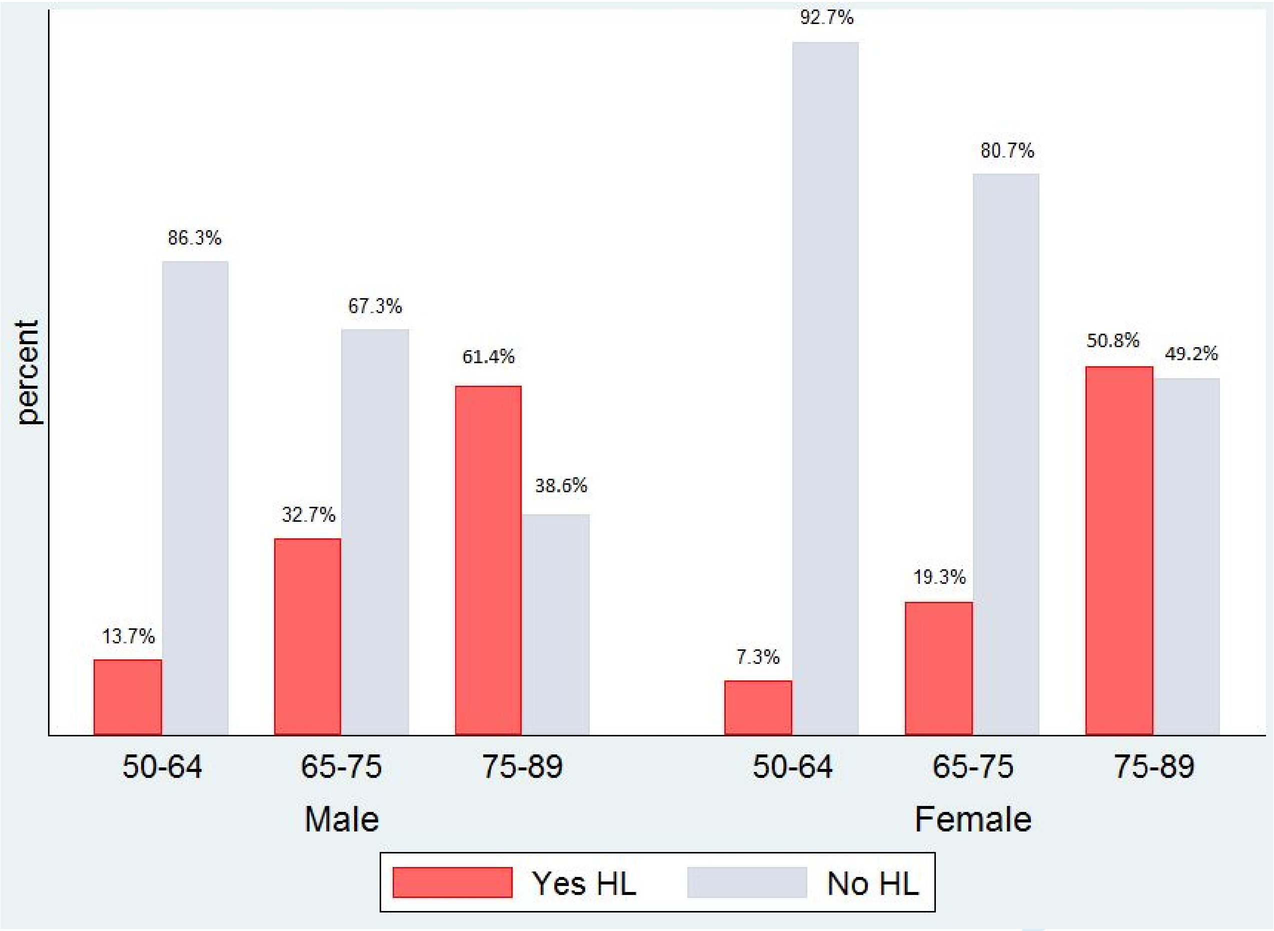

Figure 2.

Hearing loss by age group and gender (N=8,529 participants, aged 50-89, from the seventh wave of the English Longitudinal Study of Ageing (ELSA). Hearing loss was defines as >35 dB HL at 3.0 kHz, in the better-hearing ear.

For peer review only - http://bmjopen.bmj.com/site/about/guidelines.xhtml

REFERENCES

[1] WHO. (2013). Millions of People in the world have hearing loss that can be treated or prevented. *World Health Organization*, 20.

[2] Wilson, B. S., Tucci, D. L., Merson, M. H., & O'Donoghue, G. M. (2017). Global hearing health care: new findings and perspectives. *The Lancet*, *390*(10111), 2503–2515. https://doi.org/10.1016/S0140-6736(17)31073-5

[3] Stevens, G., Group, on behalf of the G. B. of D. H. L. E., Flaxman, S., Group, on behalf of the G. B. of D. H. L. E., Brunskill, E., Group, on behalf of the G. B. of D. H. L. E., … Group, on behalf of the G. B. of D. H. L. E. (2013). Global and regional hearing impairment prevalence: an analysis of 42 studies in 29 countries. *European Journal of Public Health*, *23*(1), 146–152. Retrieved from http://dx.doi.org/10.1093/eurpub/ckr176

[4] Lutman, M. E., & Spencer, H. S. (1990). Occupational noise and demographic factors in hearing. *Acta Oto-Laryngologica. Supplementum*, *476*, 74–84.

[5] Tsimpida, D., Kaitelidou, D., & Galanis, P. (2018). Determinants of Health-related Quality of Life (HRQoL) among Deaf and Hard of Hearing Adults in Greece: a Cross-Sectional Study. *Archives of Public Health.*, 76(55)

[6] Scholes, S., Biddulph, J., Davis, A., & Mindell, J. S. (2018). Socioeconomic differences in hearing among middle-aged and older adults: cross-sectional analyses using the Health Survey for England. *BMJ Open*, *8*(2), e019615. https://doi.org/10.1136/bmjopen-2017-019615

[7] Kawachi, I., Subramanian, S. V., & Almeida-Filho, N. (2002). A glossary for health inequalities. *Journal of Epidemiology & Community Health*, *56*(9), 647-652.

[8] Marmot, M. G., Allen, J., Goldblatt, P., Boyce, T., McNeish, D., Grady, M., & Geddes, I. (2010). Fair society, healthy lives: Strategic review of health inequalities in England post-2010.

[9] Galobardes, B., Shaw, M., Lawlor, D. A., Lynch, J. W., & Smith, G. D. (2006). Indicators of socioeconomic position (part 1). *J Epidemiol Community Health*, *60*. https://doi.org/10.1136/jech.2004.023531

[10] Health Profile for England. (2018).
Retrieved from https://www.gov.uk/government/publications/health-profile-for-england-2018

[11] Poortinga, W. (2007). The prevalence and clustering of four major lifestyle risk factors in an English adult population. *Preventive Medicine*, *44*(2), 124–128. https://doi.org/10.1016/j.ypmed.2006.10.006

[12] Banks, J., Blake, M., Clemens, S., Marmot, M., Nazroo, J., Oldfield, Z., Oskala, A., Phelps, A., Rogers, N., Steptoe, A. (2018). English Longitudinal Study of Ageing: Waves 0-8, 1998-2017. [data collection]. 29th Edition. UK Data Service. SN: 5050, http://doi.org/10.5255/UKDA-SN-5050-16

[13] Siemens Audiologische Technik GmbH. Hear Check Screener User Guide. PUBLICIS; 2007 [on-line]. Available at http://www.connevans.info/image/connevans/38shearcheck.pdf

[14] Davis, A., Smith, P., Ferguson, M., Stephens, D., & Gianopoulos, I. (2007). Acceptability, benefits and costs of early screening for hearing disability study tests and models.pdf. *Health Technology Assessment*, *11*(42). https://doi.org/10.3310/hta11420

[15] Bjorntorp, P., Bray, G. A., Carroll, K. K., Chuchalin, A., Dietz, W. H., Ehrlich, G. E., … Zimmet, P. (2000). Obesity : Preventing and Managing the Global Epidemic. *WHO Technical Report*

For peer review only - http://bmjopen.bmj.com/site/about/guidelines.xhtml

Series. https://doi.org/ISBN 92 4 120894 5

[16] Department of Health. (2016). UK Chief Medical Officers' Low Risk Drinking Guidelines, (August), 11. Retrieved from https://www.gov.uk/%0Ahttps://www.gov.uk/government/uploads/system/uploads/attachment_data/file/545937/UK_CMOs__report.pdf

[17] StataCorp (2015). Stata Statistical Software: Release 14. College Station, TX: StataCorp LP.

[18] Benova, L., Campbell, O. M., Sholkamy, H., & Ploubidis, G. B. (2014). Socio-economic factors associated with maternal health-seeking behaviours among women from poor households in rural Egypt. *International journal for equity in health*, *13*(1), 111.

[19] Sung, J., Sim, C., Lee, C.-R., Yoo, C.-I., Lee, H., Kim, Y., & Lee, J. (2013). Relationship of cigarette smoking and hearing loss in workers exposed to occupational noise. *Annals of Occupational and Environmental Medicine*, *25*(1), 8. https://doi.org/10.1186/2052-4374-25-8

[20] Andrade, F. C. D., & Lopez-Ortega, M. (2017). Educational Differences in Health Among Middle-Aged and Older Adults in Brazil and Mexico. *Journal of Aging and Health*, 898264317705781. https://doi.org/10.1177/0898264317705781

[21] Martin, L. G., Schoeni, R. F., Andreski, P. M., & Jagger, C. (2012). Trends and inequalities in late-life health and functioning in England. *Journal of Epidemiology and Community Health*, *66*(10), 874–880. https://doi.org/10.1136/jech-2011-200251

[22] Zhan, W., Cruickshanks, K. J., Klein, B. E. K., Klein, R., Huang, G.-H., Pankow, J. S., … Tweed, T. S. (2011). Modifiable determinants of hearing impairment in adults. *Preventive Medicine*, *53*(4–5), 338–342. https://doi.org/10.1016/j.ypmed.2011.08.012

[23] Pierre, P. V., Fridberger, A., Wikman, A., & Alexanderson, K. (2012). Self-reported hearing difficulties, main income sources, and socio-economic status; a cross-sectional population-based study in Sweden. *BMC Public Health*, *12*, 874. https://doi.org/10.1186/1471-2458-12-874

[24] Rosenhall, U., Jonsson, R., & Soderlind, O. (1999). Self-assessed hearing problems in Sweden: a demographic study. *Audiology : Official Organ of the International Society of Audiology*, *38*(6), 328–334.

[25] Davis, A. C. (1989). The prevalence of hearing impairment and reported hearing disability among adults in Great Britain. *International Journal of Epidemiology*, *18*(4), 911–917.

[26] Cruickshanks, K. J., Wiley, T. L., Tweed, T. S., Klein, B. E. K., Klein, R., Mares-Perlman, J. A., & Nondahl, D. M. (1998a). Prevalence of Hearing Loss in Older Adults in Beaver Dam, Wisconsin: The Epidemiology of Hearing Loss Study. *American Journal of Epidemiology*. https://doi.org/10.1093/oxfordjournals.aje.a009713

[27] Mamo, S. K., Nieman, C. L., & Lin, F. R. (2016). Prevalence of Untreated Hearing Loss by Income among Older Adults in the United States. *Journal of Health Care for the Poor and Underserved*, *27*(4), 1812–1818. https://doi.org/10.1353/hpu.2016.0164

[28] Tsimpida, D., Kaitelidou, D., & Galanis, P. (2018). Barriers to the Use of Health Services among Deaf and Hard of Hearing Adults in Greece: a Cross-Sectional Study. *European Journal for Person Centred Healthcare*, 6(4) http://dx.doi.org/10.5750/ejpch.v6i4.1566

[29] Gopinath, B., Flood, V. M., McMahon, C. M., Burlutsky, G., Smith, W., & Mitchell, P. (2010). The effects of smoking and alcohol consumption on age-related hearing loss: the Blue

For peer review only - http://bmjopen.bmj.com/site/about/guidelines.xhtml

Mountains Hearing Study. *Ear Hear*, *31*. https://doi.org/10.1097/AUD.0b013e3181c8e902
[30] Üçler, R., Turan, M., Garça, F., Acar, İ., Atmaca, M., & Çankaya, H. (2016). The association of obesity with hearing thresholds in women aged 18–40 years. *Endocrine*, *52*(1), 46–53. https://doi.org/10.1007/s12020-015-0755-y
[31] Curhan, S. G., Eavey, R., Wang, M., Stampfer, M. J., & Curhan, G. C. (2013). Body mass index, waist circumference, physical activity, and risk of hearing loss in women. *American Journal of Medicine*, *126*(12), 1142.e1-1142.e8. https://doi.org/10.1016/j.amjmed.2013.04.026
[32] Bishop, C. (2012). The Ear is a Window to the Heart: A Modest Argument for a Closer Integration of Medical Disciplines. *Otolaryngology*, *02*(04), 4172. https://doi.org/10.4172/2161-119X.1000e108
[33] WHO (2015). Fact sheets: non-communicable diseases
Retrieved from https://www.who.int/topics/noncommunicable_diseases/factsheets/en/
[34] Gochman, D. S. (Ed.). (2013). *Handbook of health behavior research II: provider determinants*. Springer Science & Business Media.

For peer review only

BMJ Open

Socioeconomic and Lifestyle Factors Associated with Hearing Loss in Older Adults: A Cross-sectional Study of the English Longitudinal Study of Ageing (ELSA)

Journal:	*BMJ Open*
Manuscript ID	bmjopen-2019-031030.R1
Article Type:	Research
Date Submitted by the Author:	16-Jul-2019

Complete List of Authors:	Tsimpida, Dialechti; University of Manchester, Centre for Primary Care and Health Services Research, Division of Population Health, School of Health Sciences, Faculty of Biology, Medicine and Health<br>Kontopantelis, Evangelos; University of Manchester, Division of Informatics, Imaging & Data Sciences, School of Health Sciences, Faculty of Biology, Medicine and Health, The University of Manchester<br>Ashcroft, Darren; University of Manchester, Centre for Pharmacoepidemiology and Drug Safety, Division of Pharmacy and Optometry, School of Health Sciences, Faculty of Biology, Medicine and Health, The University of Manchester<br>Panagioti, Maria; University of Manchester, Centre for Primary Care and Health Services Research, Division of Population Health, School of Health Sciences, Faculty of Biology, Medicine and Health

**Primary Subject Heading**:	Public health
Secondary Subject Heading:	Public health
Keywords:	PUBLIC HEALTH, Hearing loss, socioeconomic position, hearing health inequalities, healthy ageing, social epidemiology

SCHOLARONE™
Manuscripts

TITLE PAGE

Socioeconomic and Lifestyle Factors Associated with Hearing Loss in Older Adults: A Cross-sectional Study of the English Longitudinal Study of Ageing (ELSA)

Dialechti Tsimpida,*
NIHR Doctoral Research Fellow, Centre for Primary Care and Health Services Research, Division of Population Health, School of Health Sciences, Faculty of Biology, Medicine and Health, The University of Manchester, Manchester, UK
** Corresponding author: Centre for Primary Care and Health Services Research,*
5th floor Williamson Building, The University of Manchester,
Oxford Road, M139PL, Manchester, UK
Tel.: +44 161 275 7006
E-mail address: dialechti.tsimpida@manchester.ac.uk

Professor Evangelos Kontopantelis,
Division of Informatics, Imaging & Data Sciences, School of Health Sciences, Faculty of Biology, Medicine and Health, The University of Manchester, Manchester, UK
E-mail address: e.kontopantelis@manchester.ac.uk

Professor Darren Ashcroft,
Division of Pharmacy and Optometry, School of Health Sciences, Faculty of Biology, Medicine and Health, The University of Manchester, Manchester, UK
E-mail address: Darren.Ashcroft@manchester.ac.uk

Dr Maria Panagioti,
Senior Lecturer, Centre for Primary Care and Health Services Research, Division of Population Health, School of Health Sciences, Faculty of Biology, Medicine and Health, The University of Manchester, Manchester, UK
E-mail address: maria.panagioti@manchester.ac.uk

Keywords: Hearing loss; socioeconomic position; hearing health inequalities; healthy ageing; social epidemiology

Word count for the abstract; **300**

Manuscript word count; **4,088**

Number of references; **39**

For peer review only - http://bmjopen.bmj.com/site/about/guidelines.xhtml

ABSTRACT

Objectives: Aims were (a) to examine whether socioeconomic position (SEP) is associated with hearing loss (HL) among older adults in England and (b) whether major modifiable lifestyle factors (high body mass index, physical inactivity, tobacco consumption and alcohol intake above the low risk level guidelines) are associated with HL after controlling for the effects of non-modifiable demographic factors and SEP.

Setting: We used data from the wave 7 of the English Longitudinal Study of Aging (ELSA), which is a longitudinal household survey dataset of a representative sample of people aged 50 and older.

Participants: The final analytical sample was 8,529 participants aged 50-89 that gave consent to have their hearing acuity objectively measured by a screening audiometry device and did not have any ear infection.

Primary and secondary outcome measures: HL defined as >35 dBHL at 3.0 kHz (better-hearing ear). Those with HL were further subdivided into two categories depending on the number of tones heard at 3.0kHz.

Results: HL was identified in 32.1% of men and 22.3% of women aged 50-89. Those in a lower SEP were up to two times more likely to have HL; the adjusted odds of HL were higher for those with no qualifications versus those with a degree/higher education, those in routine/manual occupations versus those in managerial/professional occupations, and those in the lowest versus the highest income and wealth quintiles. All regression models showed that socioeconomic and the modifiable lifestyle factors were strongly associated with HL after controlling for age and gender.

Conclusions: Socioeconomic and lifestyle factors are associated with HL among older adults as strongly as core demographic risk factors, such as age and gender. Socioeconomic inequalities and modifiable lifestyle behaviours need to be targeted by health policy strategies, as an important step in designing interventions for individuals that face hearing health inequalities.

For peer review only - http://bmjopen.bmj.com/site/about/guidelines.xhtml

Strengths and limitations of this study

- The first study that focuses on modifiable lifestyle factors (such as high body mass index, physical inactivity, tobacco consumption and alcohol intake above the low risk level guidelines) associated with HL among older adults in England.
- Examines the effects of four different SEP indicators to HL (education, occupation, income, wealth), instead of a proxy measure to reflect one's total SEP, capturing therefore most of the variation in socioeconomic stratification, to the objectively measured HL in older adults.
- The analyses were based on a representative cohort of 8,529 participants contained in ELSA, which is a rich resource of information on the dynamics of health, social, wellbeing and economic circumstances of the English population aged 50 and over.
- The ELSA dataset did not contain information concerning the occupational and social noise exposure, which has a damaging effect in hearing, but we examined the association of manual occupations with HL and its attenuation by modifiable determinants including smoking habit, which is of a higher prevalence among those that work in routine and manual occupations in England.
- All the analysed factors explained less than one third of the variance for the occurrence of HL suggesting that there are additional major factors associated with HL in older adults which have not been included in our analyses.

For peer review only - http://bmjopen.bmj.com/site/about/guidelines.xhtml

INTRODUCTION

Hearing loss (HL) is a major global health challenge and the most prevalent sensory disorder. Approximately 15% of the global adult population has some degree of HL (of at least ≥25 dB HL in the better-hearing ear)[1] and almost 7% has disabling HL (defined as a hearing threshold ≥40 dB HL in the better ear)[2]. HL has been associated with negative physical, social, cognitive, economic and emotional consequences and is the fourth leading contributor to years lived with disability worldwide[2].

Previous studies have reported that HL increases with age[3], exposure to high occupational and social noise[4] and occurs more commonly among men[3]. There is growing evidence that there are a number of modifiable risk factors resulting in the aetiology of HL[5,6] and, if eliminated, might prevent more than the half cases of HL[2]. It can thus be suggested that there is a high potential for reducing the burden of HL, if we understand the modifiable factors and the mechanisms that lead to hearing health inequalities, which -following the glossary for health inequalities[7]- could be defined as the *avoidable differences in people's hearing health across different social and/or population groups*.

Prior research has established health disparities in a wide range of health conditions according to socioeconomic position (SEP)[8]. Furthermore, there is evidence that several modifiable lifestyle factors, such as smoking[9], alcohol consumption[10], high body mass index and physical inactivity[11] are associated with hearing health. Of course, the causal paths are not clear, and these associations may be confounded by deprivation or aspects of deprivation (e.g. type of occupation). Nevertheless, quantifying such associations is the first step in that direction; hearing health inequalities is an emerging research area and the existing evidence on the relationship of HL with SEP and modifiable lifestyle factors is limited. There is a major public health need to assess whether HL is associated with SEP and lifestyle factors because this understanding could inform recommendations for HL preventative strategies. These could include wider implementation of interventions to promote 'healthier lifestyles', or governmental policies for socioeconomic equity among older people in the community.

For peer review only - http://bmjopen.bmj.com/site/about/guidelines.xhtml

The aims of this study were (a) to examine whether SEP is associated with HL among older adults in England and (b) whether major modifiable lifestyle factors are associated with HL after controlling for the effects of non-modifiable demographic factors and SEP. This study is the first that examines the effects of four different SEP indicators (education, occupation, income, wealth), encompassing thus aspects of the life-course socioeconomic stratification[12], to the objectively measured HL in older adults. In addition, is the first study that explores how major lifestyle factors for general health outcomes in the English population aged 50 years old and above (such as smoking, high BMI, insufficient physical activity, tobacco consumption and alcohol intake above the low risk level guidelines)[13,14] account for the variance in HL.

METHODS

Study population

The present study used data from the English Longitudinal Study of Aging (ELSA). The ELSA is a longitudinal household survey dataset of a representative sample of people aged 50 and older in England. It is designed as a large-scale prospective cohort study, with repeat measures of core variables over numerous waves, in order to explore trajectories on the health, social, wellbeing and economic circumstances.[15]The current sample contains data from up to eight waves of data collection covering a period of 15 years, with an ongoing two-year follow-up longitudinal design.[16]

Objective hearing health data was available only in wave 7, where information was collected from 9,666 participants, between June 2014 and May 2015. For the purposes of this study, the final analytical sample was n=8,529 participants, aged 50-89, that gave consent to have their hearing acuity measured by a screening audiometry device and did not have any ear infection or a cochlear implant.

Patient and Public Involvement

For peer review only - http://bmjopen.bmj.com/site/about/guidelines.xhtml

Patients were not involved in the conduct of the study.

Hearing test

A handheld audiometric screening device (HearCheck™)[17] was used for the objective measurement of hearing acuity. This is a portable and easy-to-use hearing screening test by Siemens, that tests for audibility of pure tone beeps, according to the number of tones that the respondent can hear for each sequence (at 1.0 kHz and 3.0 kHz), per each ear. The functional test sequence begins with a series of three sounds, that have decreasing volume at 1.0 kHz (55 dB HL, 34 dB HL, 20 dB HL) and afterwards another three sounds with decreasing volume at 3.0 kHz (75 dB HL, 55 dB HL, 35 dB HL). Prerequisites for the test were the device to make proper contact with the ear that is tested, hearing aid(s), glasses, earrings and hair bands to be removed to prevent from getting in the way of the hearing device and the room to be as quiet as possible. Participants indicated when they hear the sound by raising their finger. The total number of tones that the participants indicated they could hear in the sequence of sounds at 1.0 kHz and 3.0. kHz, per each ear, was recorded and the total tones heard in the better-hearing ear used for the categorization of those with HL.

Previous studies have assessed the accuracy of the Siemens HearCheck™ in detecting hearing loss and compared it with pure tone air conduction averages designated as gold standard values. Fellizan-Lopez *et al.* (2011) found that in cases of moderate or worse hearing loss, the HearCheck™ test fulfils all criteria of high sensitivity rate, high specificity rate and high positive predictive values to be considered an accurate tool to screen for hearing loss, without the need for soundproof audiometry booths[18].

Outcomes

Hearing loss

For peer review only - http://bmjopen.bmj.com/site/about/guidelines.xhtml

HL was defined as >35 dB HL at 3.0 kHz, in the better-hearing ear. Those with HL were further subdivided into two categories depending on the number of tones heard at 3.0 kHz. This is the level where intervention for HL has been shown as definitely beneficial.[19] For that reason this categorisation has previously been used in the literature for the characterisation of those assessed by the same audiometric screening device (HearCheck™)[6]. Thus, we further explored potential differences in the association between SEP indicators and HL, according to the severity of HL, as measured by HearCheck™. The categorization of those with HL was as following:

(a) "*Moderate HL*": tones heard at 75 dB HL and 55 dB HL but not at 35 dB HL (the first 2 of the three tones at 3.0 kHz heard),

(b) "*Moderately severe or severe HL*": tone heard or not at 75 dB HL and tones not heard at 55 dB HL and 35 dB HL (0 or 1 of the three tones at 3.0 kHz heard).

The ordinal variable "hearing acuity" (in the better-hearing ear) was consisted of the above two categories of HL and the category of "normal hearing", which was defined as having heard all the three tones of the hearing screening test at 3.0 kHz.

Indicators of socioeconomic position

Education, occupation, income and wealth were the four selected indicators of SEP and information was collected in the seventh wave of ELSA, between June 2014 and May 2015. We considered five categories of the highest educational attainment: degree/higher education; A level (Level 3 of the National Qualifications Framework); O levels CSE (Certificate of Secondary Education); foreign/other; no qualifications. Tertiles of self-reported occupation were based on the National Statistics socio-economic classification (NS-SEC): managerial and professional; intermediate; routine and manual occupations). The relative financial position of the participants was captured by quintiles of the net household income (first quintile lowest; fifth quintile highest) that is summed across household members. In order to avoid the information bias that is related to the retirement status, we used quintiles of the total non-pension wealth

For peer review only - http://bmjopen.bmj.com/site/about/guidelines.xhtml

that is reported at the household level (first quintile lowest; fifth quintile highest), which represents the sum of net financial wealth, net physical wealth and net housing wealth.

Covariates

Age, marital status, retirement status and non-medical determinants of health (body mass index, physical activity, tobacco and alcohol consumption) were assessed as covariates in the association between SEP indicators and HL[5].

Age was categorised into three groups (50-64, 65-74, 75-89), to allow for a comparison with Benova et al.,[20] who examined the association of socioeconomic position with hearing difficulty in ELSA wave 2.

Marital status was dichotomised into those that are currently married or not. Those who are currently married included the categories a) married, first and only marriage, b) in a registered Civil Partnership, c) remarried, in a second or later marriage. Those that categorised as not currently married included the categories a) single, that is never married and never registered in a marriage, b) separated, but still legally married, c) divorced, d) widowed.

Retirement status was dichotomised into those who were retired or not, according to the self-reported employment status.

Body Mass Index (BMI) measurements were grouped in four categories, according to WHO definitions[21]: (a) underweight: BMI under 18.5, (b) normal: BMI 18.5 or over but less than 25, (c) overweight: BMI 25 or over but less than 30, and (d) obese: BMI 30 or over.

Tobacco consumption of any type of nicotine products was recoded into three categories: those that were current smokers, those that were former smokers and those that never smoked. Both current and former smokers answered the question of 'number of cigarettes smoked per day', to explore whether they were occasional or regular smokers.

Alcohol consumption was recorded using several continuous variables such as the number of days of alcohol consumption in the last seven days and the number of (a) measures of spirit, (b) glasses of wine and (c) pints of beer that the respondents had consumed during this period. We constructed a continuous variable to represent the sum of units of alcohol that

For peer review only - http://bmjopen.bmj.com/site/about/guidelines.xhtml

the participants consumed in the last seven days, according to the Chief Medical Officer's Drinking Guidelines[22], that counts as 1 unit each measure of spirit and as 2 units each glass of wine of pint of beer. The constructed variable of units of alcohol during the last seven days was further dichotomised into those that consumed more than 14 units of alcohol the last seven days or not, in a separate variable.

Levels of physical activity were described by three ordinal variables that examined the frequency that the respondents do rigorous, moderate or mild sports or activities, with possible answers (a) more than once a week, (b) once a week, (c) one to three times a month and (d) hardly ever, or never.

Statistical analysis

Categorical variables are presented as absolute (n) and relative (%) frequencies, while continuous variables are presented using their mean and standard deviation. The Kolmogorov-Smirnov test and normal plots were used to test the normality of the quantitative variable distributions. All the 8,529 individuals (of the 9,666 initial sample in ELSA wave 7), had usable objective hearing data, measured by a qualified nurse. In total, 257 participants refused to have the assessment (the 2.6% of the full cohort of 9,666 participants). As there was no pattern in the missing data regarding age, sex, education, occupation, income and wealth and due to low proportion of missingness (<5%), records with missing data were dropped from the analyses.

We fitted multiple logistic regression models to evaluate the odds of HL at various socioeconomic strata, controlling for gender, age and non-medical determinants of health (BMI, physical activity, tobacco and alcohol consumption). Additionally, we fitted four separate stepwise logistic regression models, to examine the association of HL with non-modifiable (age, gender: Step 1), partly modifiable (education, occupation, income, wealth: Step 2, respectively), and fully modifiable lifestyle risk factors (body mass index, physical activity, tobacco and alcohol consumption: Step 3). Age was entered into the multivariable logistic regression models as a continuous variable, to maximise power.

For peer review only - http://bmjopen.bmj.com/site/about/guidelines.xhtml

The variants of pseudo R squared statistics were based on the deviance of the models and used to express how much variance in the outcome is explained by the variables in each stepwise multiple logistic regression model. The variance inflation factor (VIF) was used as an indicator of multicollinearity and the Hosmer-Lemeshow test was used as a post estimation tool, which quantified the goodness-of-fit of the models. For all models, odds ratios, 95% confidence intervals, unadjusted and adjusted coefficients' beta values, pseudo R^2 and mean VIFs are presented. The two-tailed significance level was set ≤0.05. All data were analysed using Stata version 14 (StataCorp, 2015)[23].

RESULTS

Socio-demographic characteristics

Overall, 26.6% (2,266/8,529) of adults aged 50-89 had HL >35 dB HL at 3.0 kHz. The percentages were 32.1% (1,198/3,728, 95%CI 0.31 to 0.34) for men and 22.3% (1,068/4,801, 95%CI 0.21 to 0.23) for women, respectively. Table 1 shows the distribution of socio-demographic characteristics of the sample (n=8,529, aged 50-89) according to hearing acuity. The proportion of men and women with HL >35 dB HL at 3.0kHz was 53.9 (1,158) and 46.2 (994), respectively. However, men were 1.5 times more likely to have moderately severe or severe HL compared to women. One in three adults aged 65-75 had hearing loss and the percentage of HL in age band 75-89 was threefold larger than in age band 50-64, as one out of every two adults aged 75-89 had HL >35 dB HL at 3.0kHz.

Table 1

Lifestyle factors

Lifestyle factors of the participants are presented in Table 2. Over half of the participants were current or former smokers. In addition, patterns of high levels of alcohol consumption

For peer review only - http://bmjopen.bmj.com/site/about/guidelines.xhtml

among all participants were revealed, with average consumption of more than 14 units of alcohol in the last seven days for two out of three participants (5,223/8,528, 95%CI 0.60 to 0.61). Nearly one out of every three of those drinking above the low risk level guidelines[22] (1,457/5.223, 95%CI 0.27 to 0.29) had HL >35 dB HL at 3.0 kHz.

Three out of four of those with HL >35 dB HL at 3.0 kHz were overweight or obese. Furthermore, those with HL >35 dB HL at 3.0 kHz were twice as likely to hardly ever or never engage in moderate or mild sports activities compared to hearing participants.

Table 2

Hearing Loss

Table 3 and Figure 1 show the results of multiple logistic regression analysis with HL >35 dB HL at 3.0 kHz as the dependent variable and SEP indicators as the independent variables, per each gender. The adjusted odds of HL were higher for those with no qualifications versus those with a degree/higher education (men: OR 1.87, 95% CI 1.47-2.38, women: OR 1.53, 95% CI 1.21-1.95), those in routine/manual occupations versus those in managerial/professional occupations (men: OR 1.92, 95% CI 1.43-2.63, women: OR 1.25, 95% CI 1.03-1.54), and those in the lowest versus the highest income and wealth quintiles (men: OR 1.62, 95% CI 1.08-2.44, women: OR 1.36, 95% CI 0.85-2.16 and men: OR 1.72, 95% CI 1.26-2.35, women: OR 1.88, 95% CI 1.37-2.58, respectively).

Table 3 & Figure 1

Table 4 shows the summary of stepwise logistic regression analysis for variables predicting HL >35 dB HL at 3.0 kHz. All regression models were statistically significant. Age and gender only explained about 15% of the variance in the likelihood of HL. The addition of lifestyle factors attenuated significantly the association between the HL and SEP indicators and

For peer review only - http://bmjopen.bmj.com/site/about/guidelines.xhtml

For peer review only

in total the addition of SEP and lifestyle factors in the regression models explained another 10 to 15% of the variance in the likelihood of HL. The total variance explained in the overall models containing demographic factors, SEP and lifestyle factors ranged between 25 and 27%. This finding suggests that SEP and lifestyle factors have an equal contribution to HL as age and gender.

The differences in hearing loss prevalence between males and females were observed across all age bands investigated. However, we noticed that the rate of deterioration of hearing acuity as age increases was similar between each age band and nearly to 60% in both genders (Figure 2). The difference in prevalence begins at the age band “50-64”, where men were twice as likely to have HL.

Table 4

DISCUSSION

Summary of main findings

In this study we examined whether SEP and modifiable lifestyle factors are associated with HL among older adults in England. We found that variation in education, occupation, income and wealth, which are important determinants of health inequality, are associated with HL. SEP was strongly associated with the likelihood of HL in older adults, with the higher levels of education, income and wealth being less likely to be associated with HL, and the manual occupations increased the likelihood of HL. We also found that socioeconomic and several modifiable lifestyle factors (such as high body mass index, physical inactivity, tobacco consumption and alcohol intake above the low risk level guidelines[22]) are associated with the likelihood of HL as strongly as well-established demographic factors such as age and gender HL. These findings suggest that a large proportion of HL burden is potentially preventable and support the proposition of Scholes et al.[6], that there is serious potential to reduce the prevalence and impacts of HL by understanding the impact of socioeconomic inequality in

For peer review only - http://bmjopen.bmj.com/site/about/guidelines.xhtml

hearing health. Thus, the incidence and severity of HL in England could be significantly reduced by governmental policies to mitigate socioeconomic disparities and public health interventions to promote healthier lifestyles in middle-aged and older adults in England. The occurrence of objective hearing data eliminated the different types of bias that occur in self-reporting hearing difficulties[24], strengthening the accuracy of findings.

Strengths and limitations

The main strength of our study was that is the first to examine the association of four separate SEP indicators with HL among older adults in England, instead of a proxy measure to reflect one's total SEP, capturing therefore most of the variation in socioeconomic stratification[12] and also the role of modifiable lifestyle risk factors in these associations. Another strength is that the analyses were based on a representative cohort of 8,529 participants contained in ELSA, which is a rich resource of information on the dynamics of health, social, wellbeing and economic circumstances in the English population aged 50 and older[16].

However there are also important limitations. First, no causal or temporal relationships can be established between lifestyle factors and HL in this cross-sectional study. Unhealthy lifestyle behaviours could lead to HL in older people but is also possible that older people adopt less healthy lifestyles after HL. Second, all the analysed factors explained less than one third of the variance for the occurrence of HL suggesting that there are additional major factors associated with HL in older adults which have not been included in our analyses. Longitudinal analyses using a broader range of physical health, mental health and social care variables are highly recommended to obtain a comprehensive understanding of modifiable factors which contribute to HL among older adults in England. Third, the ELSA dataset did not include information concerning the occupational and social noise exposure, which has a damaging effect in hearing[4]. We therefore were not able to examine the association of noise exposure with smoking in the relationship of SEP with HL, as in a previous study which found that the smoking habit in workers exposed to occupational noise greatly influenced HL[25]. However, we

For peer review only - http://bmjopen.bmj.com/site/about/guidelines.xhtml

examined the association of manual occupations with HL and its attenuation by modifiable determinants including smoking habit, which is of a higher prevalence among those that work in routine and manual occupations in England[13]. Finally, we did not use in our analyses the non-response statistical weights for the refreshment sample members, who were selected from HSE 2011 and 2012, which may have reduced the generalizability of our findings.

Research and policy implications

A number of previous studies have reported that the odds of HL in older adults were significantly increased for those with lower educational attainment [6,10,26,27], and those in manual versus non-manual occupations[28,29,30,31]. Besides, income has also been reported as a correlate of HL, with the prevalence of untreated HL being higher among low-income older adults in the United States[31]. In our study, those in the lowest quintile of net household income had disproportionally higher percentages of moderate HL compared to moderately severe or severe HL, but this pattern was not revealed in the quintiles of wealth, as expected. This may reveal a possible delay in diagnosis of hearing problems among those in lower SEP due to financial barriers in access to health services[32], which needs further exploration, as HL is highly undiagnosed and untreated among older adults in England[20].

International studies have also shown that tobacco consumption, high body mass and high fat and high calorie food consumption can have an extensive impact on hearing [11,33,34,35] On the other hand, a higher level of physical activity is related inversely with risk of HL[34]. In our study, two out of three of the participants were drinking more than the low risk level of the 14 units of alcohol a week[22]. We considered therefore that alcohol consumption above the low risk level guidelines may play an important role in the association between SEP and HL among the English population and thus we included this variable in the regression models, which has not been previously examined in the literature for the English population. Our findings showed that drinking above the low risk level guidelines increased the likelihood of HL, being in line with Chief Medical Officer's Drinking Guidelines[22], which suggest that *it is safest not to drink regularly more than 14 units per week, to keep health risks from drinking alcohol to a low level*.

For peer review only - http://bmjopen.bmj.com/site/about/guidelines.xhtml

The associations between indicators of lower socioeconomic position and hearing loss may be markers of less healthy lifestyle[5], which may explain the link between HL and socioeconomic and lifestyle factors investigated. Cruickshanks et al, (2015) [36] addressed hearing loss in a younger population-based sample (aged 18 to 74 years) of Hispanics/Latinos and included in the multivariable-adjusted model of their study the body mass index, smoking, and alcohol but they found that these factors were not significantly associated with hearing impairment. That may reveal that hearing loss in older population (e.g. 50 years and above) is probably associated with different risk factors or even with the cumulative effect of the socioeconomic and lifestyle risk factors across the life-course.

The higher prevalence of HL among men aged 50 and above compared to women has also been reported in other studies[3,6]. However, we noticed that the rate of deterioration of hearing acuity as age increases was similar between each age band and nearly to 60% in both genders, as Figure 2 shows. The difference in prevalence begins at the age band “50-64”, where men were twice as likely to have HL. Thus, the differences in modifiable lifestyle factors that were revealed in the stepwise regression models may finally explain why the male sex is often cited as consistent risk factor for hearing loss[35,36,37], leading to the exploration of modifiable determinants that are common in both genders[5] and paving the way for interventions to improve the population’s hearing health.

In terms of policy, providing evidence concerning the critical variables associated with HL is an important step in designing targeted services and interventions for individuals that face hearing health inequalities, and especially of those in the lowest SEP groups, were the burden of HL fall highest. This is of major importance for the population in England, as the sensor diseases are the first leading cause of morbidity among adults 70 years and older and the second leading cause among adults 50-69 years[13]. Our findings provide support to the view that HL is a non-communicable disease[38] which can be prevented or ameliorated by governmental policies to mitigate socioeconomic disparities and public health interventions to promote healthier lifestyles in middle-aged and older adults in England.

For peer review only - http://bmjopen.bmj.com/site/about/guidelines.xhtml

Conclusion

The main finding of our study is that HL is strongly associated with socioeconomic factors and modifiable lifestyle behaviours. Our findings are supportive of a new conceptualisation of HL which argues that HL is not necessarily an inevitable accompaniment of ageing, but also a potential preventable lifestyle disease, paving the way for the term *lifestyle-related hearing loss*, where *lifestyle* refers to *social practices and ways of living adopted by individuals that reflect personal, group, and socio-economic identities*[39], instead of the non-inclusive term "*age-related hearing loss*". Future research in hearing health inequalities should investigate the role of the prolonged exposure to these modifiable lifestyle behaviours in the development of HL and the role of other comorbid chronic diseases in the elderly.

For peer review only

Contributors DT, EK, DA and MP were responsible for developing the design of the study. DT was responsible for conducting the analyses, interpreting the results and drafting the manuscript. DT, EK, DA and MP critically revised the manuscript. All authors have read and approved the final manuscript.

Acknowledgment DT would like to acknowledge the contribution of Dr Piers Dawes and Prof Neil Pendleton, who had provided assistance in obtaining funding and were the former supervisors over her NIHR Manchester Biomedical Research Centre PhD Studentship, but did not fulfil the criteria set out in the Authorship Guidelines of The University of Manchester to be listed as authors.

Funding This research was funded by the NIHR Manchester Biomedical Research Centre (BRC). The views expressed are those of the authors and not necessarily those of the BRC, the NIHR or the Department of Health.

Competing interests None declared

Patient consent Not required

Ethics approval Not required. The study used data from the English Longitudinal Study of Ageing (ELSA).

Provenance and peer review Non commissioned; externally peer reviewed

Data sharing statement The English Longitudinal Study of Ageing dataset is available via the UK Data Service (http://www.ukdataservice.ac.uk). Statistical code is available from the corresponding author at dialechti.tsimpida@manchester.ac.uk.

Open Access This is an Open Access article distributed in accordance with the Creative Commons Attribution Non Commercial (CC BY-NC 4.0) license, which permits others to distribute, remix, adapt, build upon this work non-commercially, and licence their derivative works on different terms, provided the original work is properly cited and the use is non-commercial. See: http://creativecommons.org/licenses/by-nc/4.0/

© Article author(s) (or their employer(s) unless otherwise stated in the text of the article) 2019. All rights reserved. No commercial use is permitted unless otherwise expressly granted.

For peer review only - http://bmjopen.bmj.com/site/about/guidelines.xhtml

Table 1
Participants socio-demographic characteristics (N=8,529, aged 50-89)

**Variable**	Hearing acuity % (N) in the better-hearing ear			
	**Normal Hearing**	**HL >35 dB HL at 3.0kHz**	**Moderate HL***	**Moderately severe or severe HL****
**Gender**				
Male	40.7 (2,488)	53.9 (1,158)	50.1 (727)	61.5 (431)
Female	59.3 (3,623)	46.2 (994)	49.9 (724)	38.5 (270)
**Age**[a]	64.3 (9.29)	69.7 (19.19)	70.0 (15.85)	69.1 (24.41)
**Age group**				
50-64	51.3 (3,135)	16.2 (349)	19.3 (280)	9.8 (69)
65-74	34.5 5 (2,108)	33.6 (722)	36.9 (535)	26.7 (187)
75-89	14.2 (868)	50.2 (1,081)	43.8 (636)	63.5 (445)
**Currently married**				
No	31.2 (1,908)	38.4 (826)	37.5 (544)	40.2 (282)
Yes	68.8 (4,202)	61.6 (1,326)	62.5 (907)	59.8 (701)
**Retirement status**				
Retired	52.4 (3,205)	78.3 (1,685)	76.6 (1,112)	81.3 (573)
Not retired	47.6 (2,905)	21.7 (467)	23.4 (339)	18.3 (128)
**Education**				
Degree/Higher Education	33.7 (1,996)	26.4 (562)	28.1 (404)	22.9 (158)
A level	10.0 (596)	6.4 (137)	7.0 (100)	5.4 (37)
O level/CSE grade	24.4 (1,448)	22.3 (473)	22.4 (321)	22.0 (152)
Foreign/Other	13.5 (798)	11.9 (252)	11.9 (171)	11.7 (81)
No qualifications	18.4 (1,090)	33.0 (701)	30.6 (439)	38.0 (262)
**Occupation based National Statistics Socio-economic Classification (NS-SEC)**				
Managerial and professional occupations	23.4 (1,158)	21.5 (423)	21.6 (285)	21.2 (138)
Intermediate occupations (non-manual)	43.4 (2,149)	33.8 (665)	36.2 (477)	28.9 (188)
Routine and manual occupations	33.2 (1,644)	44.7 (1,643)	42.2 (1,318)	49.9 (325)

(Continued)

For peer review only - http://bmjopen.bmj.com/site/about/guidelines.xhtml

Table 1 (Continued)

Participants socio-demographic characteristics (N=8,529, aged 50-89)

Variable	Hearing acuity % (N) in the better-hearing ear: Normal Hearing	HL >35 dB HL at 3.0kHz	Moderate HL*	Moderately severe or severe HL**
**Net Household Income**				
First quintile (lowest)	17.0 (872)	21.3 (421)	19.7 (262)	24.8 (159)
Second quintile	18.7 (959)	24.8 (489)	24.7 (329)	24.9 (160)
Third quintile	20.1 (1,034)	23.0 (453)	22.3 (297)	24.3 (156)
Fourth quintile	22.5 (1,154)	18.6 (367)	19.9 (265)	15.9 (102)
Fifth quintile (highest)	21.7 (1,112)	12.3 (243)	13.4 (178)	10.1 (65)
**Net Financial Wealth**				
First quintile (lowest)	15.5 (794)	14.7 (290)	14.9 (199)	14.2 (91)
Second quintile	17.1 (879)	24.1 (475)	22.1 (294)	28.2 (181)
Third quintile	19.6 (1,006)	23.6 (466)	23.4 (311)	24.1 (155)
Fourth quintile	23.5 (1,204)	20.3 (400)	21.3 (284)	18.1 (116)
Fifth quintile (highest)	24.3 (1,248)	17.3 (342)	18.3 (243)	15.4 (99)

Values are expressed as % (N) unless otherwise is indicated.

[a]Mean (Standard deviation)

*Moderate hearing loss: tones heard at 75 dB HL and 55 dB HL but not at 35 dB HL (the first **2 of the three tones at 3.0 kHz** heard)

Moderately severe or severe hearing loss: tone heard or not at 75 dB HL and tones not heard at 55 dB HL and 35 dB HL (0 or 1 of the three tones at 3.0 kHz** heard).

For peer review only - http://bmjopen.bmj.com/site/about/guidelines.xhtml

Table 2
Participants' lifestyle factors (N=8,529, aged 50-89)

**Variable**	**Hearing acuity % (N) in the better-hearing ear**			
	**Normal Hearing**	**HL >35 dB HL at 3.0kHz**	**Moderate HL***	**Moderately severe or severe HL****
**Tobacco consumption (any type of nicotine products)**				
Current	11.7 (712)	10.0 (215)	9.6 (139)	10.8 (76)
Former	49.0 (2,996)	56.7 (1,219)	55.8 (810)	58.4 (409)
Number of cigarettes smoked per day [a]	12.79 (14)	12.79 (13)	12.69 (13)	11.90 (12)
Never	39.3 (2,403)	33.3 (718)	34.6 (502)	30.8 (216)
**Alcohol consumption (in the last 7 days)**				
Number of days of alcohol consumption [b]	3 (3)	3 (4)	3 (4)	3 (4)
Number of measures of spirit [a]	2.1 (2)	2.3 (3)	2.2 (3)	2.6 (3)
Number of glasses of wine [a]	4.3 (6)	3.6 (5)	3.9 (6)	3.1 (4)
Number of pints of beer [a]	2.1 (2)	2.3 (3)	2.3 (3)	2.4 (3)
Total units of alcohol in the last 7 days [a]	15.0 (18)	14.2 (19)	14.5 (21)	13.5 (17)
Consumption of more than 14 units	61.6 (3,766)	67.7 (1,457)	67.3 (977)	68.5 (480)
**BMI Classification**				
Underweight	3.4 (160)	5.0 (92)	4.9 (60)	5.3 (32)
Normal	26.9 (1,255))	20.6 (376)	19.6 (239)	22.7 (137)
Overweight	40.0 (1,869)	42.8 (780)	41.4 (506)	45.4 (274)
Obese	29.7 (1,390)	31.6 (576)	34.1 (416)	26.6 (160))
**Physical Activity**				
Frequency does rigorous sports or activities				
More than once a week	23.0 (1,407)	14.3 (307)	16.1 (233)	10.6 (74)
Once a week	10.3 (626)	7.0 (151)	7.9 (115)	5.1 (36)
One to three times a month	10.1 (617)	7.1 (153)	7.6 (111)	6.0 (42)
Hardly ever, or never	56.6 (3,459)	71.6 (1,541)	68.4 (992)	78.3 (549)

(Continued)

For peer review only - http://bmjopen.bmj.com/site/about/guidelines.xhtml

Table 2 (Continued)

Participants' lifestyle factors (N=8,529, aged 50-89)

Variable	Hearing acuity % (N) in the better-hearing ear: Normal Hearing	HL >35 dB HL at 3.0kHz	Moderate HL*	Moderately severe or severe HL**
**Physical Activity** (continued)				
Frequency does moderate sports or activities				
More than once a week	68.4 (4,180)	51.3 (1,104)	53.7 (780)	46.2 (324)
Once a week	12.6 (771)	13.6 (292)	14.1 (204)	12.6 (88)
One to three times a month	5.9 (360)	7.8 (169)	7.6 (110)	8.4 (59)
Hardly ever, or never	13.1 (799)	27.3 (587)	24.6 (357)	32.8 (230)
Frequency does mild sports or activities				
More than once a week	83.9	73.7	76.0 (1,103)	68.9 (483)
Once a week	8.2	10.1	9.8 (142)	10.5 (74)
One to three times a month	2.3	3.5	3.3 (48)	4.0 (28)
Hardly ever, or never	5.6	12.7	10.9 (158)	16.6 (116)

Values are expressed as % (N) unless otherwise is indicated.

[a]Mean (Standard deviation)

[b]Median (Range)

*Moderate hearing loss: tones heard at 75 dB HL and 55 dB HL but not at 35 dB HL (the first **2 of the three tones at 3.0 kHz** heard)

Moderately severe or severe hearing loss: tone heard or not at 75 dB HL and tones not heard at 55 dB HL and 35 dB HL (0 or 1 of the three tones at 3.0 kHz** heard).

For peer review only - http://bmjopen.bmj.com/site/about/guidelines.xhtml

Table 3.
Multiple logistic regression analysis of N=8,529, aged 50-89 with **HL >35 dB HL at 3.0kHz** in better –hearing ear as dependent variable and **SEP indicators** as independent variables

	Unadjusted OR (95% CI)*		Adjusted OR (95% CI)**	
	Men	Women	Men	Women
**Education**				
No qualifications	2.39 (1.96-2.90)	2.67 (2.20-3.24)	1.87 (1.47-2.38)	1.53 (1.21-1.95)
Foreign/Other	1.06 (0.83-1.36)	1.37 (1.07-1.74)	1.46 (1.09-1.94)	0.99 (0.74-1.32)
O level/CSE grade	1.56 (1.29-1.89)	1.00 (0.80-1.25)	1.42 (1.13-1.79)	0.94 (0.73-1.22)
A level	1.01 (0.77-1.32)	0.69 (0.50-0.97)	1.08 (0.78-1.51)	0.82 (0.56-1.21)
Degree/Higher Education (reference)				
**Occupation based National Statistics socio-economic classification (NS-SEC)**				
Routine and manual occupations	1.69 (1.39-2.08)	1.35 (1.15-1.59)	1.92 (1.43-2.63)	1.25 (1.03-1.54)
Intermediate occupations (non-manual)	1.47 (1.23-1.75)	1.54 (1.19-1.96)	1.61 (1.25-2.08)	1.35 (1.01-1.85)
Managerial and professional occupations (reference)				
**Net Household Income**				
First quintile (lowest)	1.94 (1.50-2.52)	3.04 (2.31-3.99)	1.62 (1.08-2.44)	1.36 (0.85-2.16)
Second quintile	2.12 (1.67-2.70)	3.00 (2.28-3.93)	1.31 (0.93-1.85)	1.40 (0.89-2.18)
Third quintile	1.98 (1.56-2.51)	2.31 (1.75-3.05)	1.40 (1.01-1.94)	1.08 (0.69-1.67)
Fourth quintile	1.38 (1.08-1.74)	1.65 (1.23-2.20)	1.09 (0.80-1.49)	1.08 (0.70-1.66)
Fifth quintile (highest) (reference)				
**Net Financial Wealth**				
First quintile (lowest)	1.11 (0.86-1.45)	1.79 (1.38-2.33)	1.72 (1.26-2.35)	1.88 (1.37-2.58)
Second quintile	1.92 (1.52-2.42)	2.39 (1.88-3.04)	1.66 (1.26-2.18)	1.33 (1.00-1.77)
Third quintile	1.63 (1.30-2.04)	1.95 (1.53-2.50)	1.45 (1.12-1.88)	1.41 (1.06-1.88)
Fourth quintile	1.06 (0.85-1.32)	1.48 (1.15-1.91)	0.96 (0.75-1.24)	1.26 (0.94-1.68)
Fifth quintile (highest) (reference)				

*Unadjusted odds ratio (OR) ** Odds Ratio adjusted for age, marital status, retirement status, body mass index, tobacco consumption, alcohol consumption and physical activity.

For peer review only - http://bmjopen.bmj.com/site/about/guidelines.xhtml

Table 4.

Summary of stepwise logistic regression coefficients for variables predicting **HL >35 dB HL at 3.0kHz in the better-hearing ear** (N=8,529, aged 50-89), according to different SEP indicators (education, occupation, income, wealth).

		Model A			Model B			Model C			Model D		
	**Step/Predictor**	**Step 1**	**Step 2a**	**Step 3**	**Step 1**	**Step 2b**	**Step 3**	**Step 1**	**Step 2c**	**Step 3**	**Step 1**	**Step 2d**	**Step 3**
**1**	**Non-modifiable**		(Education)			(Occupation)			(Income)			(Wealth)	
	Gender (female)	-.62***	-.59***	-.72***	-.62***	-.64***	-.68***	-.62***	-.69***	-.70***	-.62***	-.69***	-.62***
	Age	.12***	.11***	.10***	.12***	.13***	.11***	.12***	.11***	.11***	.12***	.11***	.12***
**2**	**Partly modifiable**												
2a	Education		-.15***	-.11***		-	-		-	-		-	-
2b	Occupation (manual)			-		.26***	.20***		-	-		-	-
2c	Net Household Income			-		-	-		-.14***	-.09***		-	-
2d	Net Financial Wealth			-		-	-		-	-		-.17***	-.11***
**3**	**Modifiable**												
	Smoking (current/former)			.10*			.09			.10*			.09**
	Alcohol consumption (> 14 units per week)			.24***			.19***			.17***			.18**
	Body mass index (<25)			-.05*			-.06			-.03			-.04
	Physical Activity (*rigorous* sports or activities, once or more/week)			-.14***			-.16***			-.12***			-.13***
	Physical Activity (*moderate* sports or activities, once or more/week)			-.24***			-.24***			-.24***			-.24***
	Physical Activity (*mild* sports or activities, once or more/week)			-.17***			-.15***			-.15***			-.14***
	Pseudo R^2	.15	.18	.28	.15	.19	.26	.17	.18	.29	.17	.18	.27
	Δ Pseudo R^2	-	.03	.10	-	.04	.07	-	.01	.11	-	.01	.09
	Mean VIF	-	-	1.16	-	-	1.20	-	-	1.24	-	-	1.15

$^{*}p < .05$. $^{**}p < .01$ $^{***}p < .001$

For peer review only

For peer review only - http://bmjopen.bmj.com/site/about/guidelines.xhtml

Figure 1.

Associations between socioeconomic position and hearing loss in middle aged and older adults (N=8,529, aged 50-89). Indicators of SEP were categories of the highest educational attainment (degree/higher education as a reference), tertiles of self-reported occupation based on the National Statistics Socio-economic Classification (NS-SEC) (managerial and professional as reference), quintiles of the net household income (first quintile lowest; fifth quintile highest) and quintiles of the total non-pension wealth that is reported at the household level (first quintile lowest; fifth quintile highest). Lines represent OR (outcome=hearing loss) and its 95% CI. Model A (rhombus): unadjusted. Model B (circles): adjusted for age, marital status, retirement status, body mass index, tobacco consumption, alcohol consumption and physical activity.

Figure 2.

Hearing loss by age group and gender (N=8,529 participants, aged 50-89, from the seventh wave of the English Longitudinal Study of Ageing (ELSA).

Hearing loss was defines as >35 dB HL at 3.0 kHz, in the better-hearing ear.

For peer review only - http://bmjopen.bmj.com/site/about/guidelines.xhtml

REFERENCES

[1] WHO. (2013). Millions of People in the world have hearing loss that can be treated or prevented. *World Health Organization*, 20.

[2] Wilson, B. S., Tucci, D. L., Merson, M. H., & O'Donoghue, G. M. (2017). Global hearing health care: new findings and perspectives. *The Lancet*, *390*(10111), 2503–2515. https://doi.org/10.1016/S0140-6736(17)31073-5

[3] Stevens, G., Group, on behalf of the G. B. of D. H. L. E., Flaxman, S., Group, on behalf of the G. B. of D. H. L. E., Brunskill, E., Group, on behalf of the G. B. of D. H. L. E., … Group, on behalf of the G. B. of D. H. L. E. (2013). Global and regional hearing impairment prevalence: an analysis of 42 studies in 29 countries. *European Journal of Public Health*, *23*(1), 146–152. Retrieved from http://dx.doi.org/10.1093/eurpub/ckr176

[4] Lutman, M. E., & Spencer, H. S. (1990). Occupational noise and demographic factors in hearing. *Acta Oto-Laryngologica. Supplementum*, *476*, 74–84.

[5] Tsimpida, D., Kaitelidou, D., & Galanis, P. (2018). Determinants of Health-related Quality of Life (HRQoL) among Deaf and Hard of Hearing Adults in Greece: a Cross-Sectional Study. *Archives of Public Health.*, 76(55)

[6] Scholes, S., Biddulph, J., Davis, A., & Mindell, J. S. (2018). Socioeconomic differences in hearing among middle-aged and older adults: cross-sectional analyses using the Health Survey for England. *BMJ Open*, *8*(2), e019615. https://doi.org/10.1136/bmjopen-2017-019615

[7] Kawachi, I., Subramanian, S. V., & Almeida-Filho, N. (2002). A glossary for health inequalities. *Journal of Epidemiology & Community Health*, *56*(9), 647-652.

[8] Marmot, M. G., Allen, J., Goldblatt, P., Boyce, T., McNeish, D., Grady, M., & Geddes, I. (2010). Fair society, healthy lives: Strategic review of health inequalities in England post-2010.

[9] Gopinath, B., Flood, V. M., McMahon, C. M., Burlutsky, G., Smith, W., & Mitchell, P. (2010). The effects of smoking and alcohol consumption on age-related hearing loss: the Blue Mountains Hearing Study. *Ear and hearing*, *31*(2), 277-282.

[10] Zhan, W., Cruickshanks, K. J., Klein, B. E. K., Klein, R., Huang, G.-H., Pankow, J. S., … Tweed, T. S. (2011). Modifiable determinants of hearing impairment in adults. *Preventive Medicine*, *53*(4–5), 338–342. https://doi.org/10.1016/j.ypmed.2011.08.012

[11] Curhan, S. G., Eavey, R., Wang, M., Stampfer, M. J., & Curhan, G. C. (2013). Body mass index, waist circumference, physical activity, and risk of hearing loss in women. *The American journal of medicine*, *126*(12), 1142-e1.

[12] Galobardes, B., Shaw, M., Lawlor, D. A., Lynch, J. W., & Smith, G. D. (2006). Indicators of socioeconomic position (part 1). *J Epidemiol Community Health*, *60*. https://doi.org/10.1136/jech.2004.023531

[13] Health Profile for England. (2018).
Retrieved from https://www.gov.uk/government/publications/health-profile-for-england-2018

[14] Poortinga, W. (2007). The prevalence and clustering of four major lifestyle risk factors in an English adult population. *Preventive Medicine*, *44*(2), 124–128. https://doi.org/10.1016/j.ypmed.2006.10.006

[15] Steptoe, A., Breeze, E., Banks, J., & Nazroo, J. (2012). Cohort profile: the English longitudinal study of ageing. *International journal of epidemiology*, *42*(6), 1640-1648.

For peer review only - http://bmjopen.bmj.com/site/about/guidelines.xhtml

[16] Banks, J., Blake, M., Clemens, S., Marmot, M., Nazroo, J., Oldfield, Z., Oskala, A., Phelps, A., Rogers, N., Steptoe, A. (2018). English Longitudinal Study of Ageing: Waves 0-8, 1998-2017. [data collection]. 29th Edition. UK Data Service. SN: 5050, http://doi.org/10.5255/UKDA-SN-5050-16

[17] Siemens Audiologische Technik GmbH. Hear Check Screener User Guide. PUBLICIS; 2007 [on-line]. Available at http://www.connevans.info/image/connevans/38shearcheck.pdf

[18] Fellizar-Lopez, K. R., Abes, G. T., Reyes-Quintos, M., Rina, T., Tantoco, M., & Leah, S. (2011). Accuracy of Siemens HearCheck™ Navigator as a Screening Tool for Hearing Loss. *Philippine Journal of Otolaryngology Head and Neck Surgery*, *26*(1), 10-15.

[19] Davis, A., Smith, P., Ferguson, M., Stephens, D., & Gianopoulos, I. (2007). Acceptability, benefits and costs of early screening for hearing disability study tests and models.pdf. *Health Technology Assessment*, *11*(42). https://doi.org/10.3310/hta11420

[20] Benova, L., Grundy, E., & Ploubidis, G. B. (2014). Socioeconomic position and health-seeking behavior for hearing loss among older adults in England. *Journals of Gerontology Series B: Psychological Sciences and Social Sciences*, *70*(3), 443-452.

[21] Bjorntorp, P., Bray, G. A., Carroll, K. K., Chuchalin, A., Dietz, W. H., Ehrlich, G. E., … Zimmet, P. (2000). Obesity : Preventing and Managing the Global Epidemic. *WHO Technical Report Series*. https://doi.org/ISBN 92 4 120894 5

[22] Department of Health. (2016). UK Chief Medical Officers' Low Risk Drinking Guidelines, (August), 11. Retrieved from https://assets.publishing.service.gov.uk/government/uploads/system/uploads/attachment_data/file/545937/UK_CMOs__report.pdf

[23] StataCorp (2015). Stata Statistical Software: Release 14. College Station, TX: StataCorp LP.

[24] Andrade, F. C. D., & Lopez-Ortega, M. (2017). Educational Differences in Health Among Middle-Aged and Older Adults in Brazil and Mexico. *Journal of Aging and Health*, 898264317705781. https://doi.org/10.1177/0898264317705781

[25] Sung, J., Sim, C., Lee, C.-R., Yoo, C.-I., Lee, H., Kim, Y., & Lee, J. (2013). Relationship of cigarette smoking and hearing loss in workers exposed to occupational noise. *Annals of Occupational and Environmental Medicine*, *25*(1), 8. https://doi.org/10.1186/2052-4374-25-8

[26] Martin, L. G., Schoeni, R. F., Andreski, P. M., & Jagger, C. (2012). Trends and inequalities in late-life health and functioning in England. *Journal of Epidemiology and Community Health*, *66*(10), 874–880. https://doi.org/10.1136/jech-2011-200251

[27] Pierre, P. V., Fridberger, A., Wikman, A., & Alexanderson, K. (2012). Self-reported hearing difficulties, main income sources, and socio-economic status; a cross-sectional population-based study in Sweden. *BMC Public Health*, *12*, 874. https://doi.org/10.1186/1471-2458-12-874

[28] Rosenhall, U., Jonsson, R., & Soderlind, O. (1999). Self-assessed hearing problems in Sweden: a demographic study. *Audiology : Official Organ of the International Society of Audiology*, *38*(6), 328–334.

[29] Davis, A. C. (1989). The prevalence of hearing impairment and reported hearing disability among adults in Great Britain. *International Journal of Epidemiology*, *18*(4), 911–917.

[30] Cruickshanks, K. J., Wiley, T. L., Tweed, T. S., Klein, B. E. K., Klein, R., Mares-Perlman, J. A., &

For peer review only - http://bmjopen.bmj.com/site/about/guidelines.xhtml

Nondahl, D. M. (1998a). Prevalence of Hearing Loss in Older Adults in Beaver Dam, Wisconsin: The Epidemiology of Hearing Loss Study. *American Journal of Epidemiology*. https://doi.org/10.1093/oxfordjournals.aje.a009713

[31] Mamo, S. K., Nieman, C. L., & Lin, F. R. (2016). Prevalence of Untreated Hearing Loss by Income among Older Adults in the United States. *Journal of Health Care for the Poor and Underserved*, *27*(4), 1812–1818. https://doi.org/10.1353/hpu.2016.0164

[32] Tsimpida, D., Kaitelidou, D., & Galanis, P. (2018). Barriers to the Use of Health Services among Deaf and Hard of Hearing Adults in Greece: a Cross-Sectional Study. *European Journal for Person Centred Healthcare*, 6(4) http://dx.doi.org/10.5750/ejpch.v6i4.1566

[33] Üçler, R., Turan, M., Garça, F., Acar, İ., Atmaca, M., & Çankaya, H. (2016). The association of obesity with hearing thresholds in women aged 18–40 years. *Endocrine*, *52*(1), 46–53. https://doi.org/10.1007/s12020-015-0755-y

[34] Bishop, C. (2012). The Ear is a Window to the Heart: A Modest Argument for a Closer Integration of Medical Disciplines. *Otolaryngology*, *02*(04), 4172. https://doi.org/10.4172/2161-119X.1000e108

[35] Hoffman, H. J., Dobie, R. A., Losonczy, K. G., Themann, C. L., & Flamme, G. A. (2017). Declining prevalence of hearing loss in US adults aged 20 to 69 years. *JAMA otolaryngology–head & neck surgery*, *143*(3), 274-285.

[36] Cruickshanks, K. J., Dhar, S., Dinces, E., Fifer, R. C., Gonzalez, F., Heiss, G., ... & Torre, P. (2015). Hearing impairment prevalence and associated risk factors in the Hispanic Community Health Study/Study of Latinos. *JAMA Otolaryngology–Head & Neck Surgery*, *141*(7), 641-648.

[37] Lin, F. R., Thorpe, R., Gordon-Salant, S., & Ferrucci, L. (2011). Hearing loss prevalence and risk factors among older adults in the United States. *Journals of Gerontology Series A: Biomedical Sciences and Medical Sciences*, *66*(5), 582-590.

[38] WHO (2015). Fact sheets: non-communicable diseases
Retrieved from https://www.who.int/topics/noncommunicable_diseases/factsheets/en/

[39] Gochman, D. S. (Ed.). (2013). *Handbook of health behavior research II: provider determinants*. Springer Science & Business Media.

For peer review only - http://bmjopen.bmj.com/site/about/guidelines.xhtml

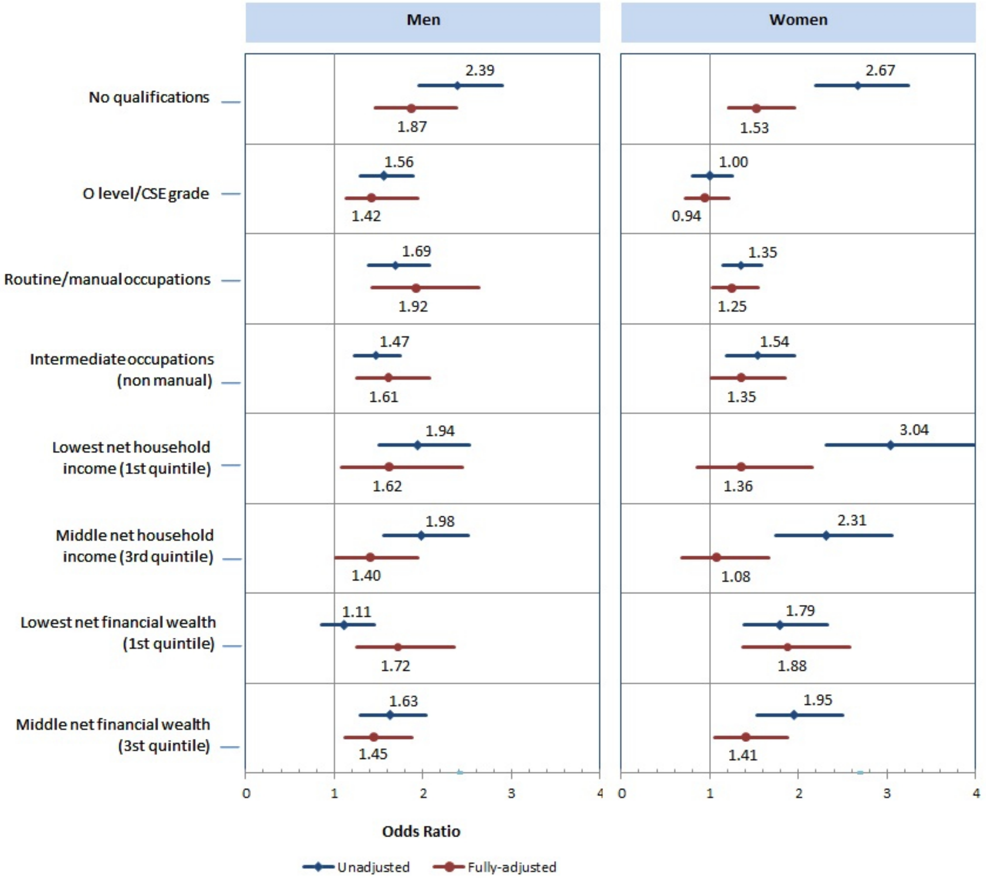

Figure 1.
Associations between socioeconomic position and hearing loss in middle aged and older adults (N=8,529, aged 50-89). Indicators of SEP were categories of the highest educational attainment (degree/higher education as a reference), tertiles of self-reported occupation based on the National Statistics Socio-economic Classification (NS-SEC) (managerial and professional as reference), quintiles of the net household income (first quintile lowest; fifth quintile highest) and quintiles of the total non-pension wealth that is reported at the household level (first quintile lowest; fifth quintile highest). Lines represent OR (outcome=hearing loss) and its 95% CI. Model A (rhombus): unadjusted. Model B (circles): adjusted for age, marital status, retirement status, body mass index, tobacco consumption, alcohol consumption and physical activity.

72x67mm (300 x 300 DPI)

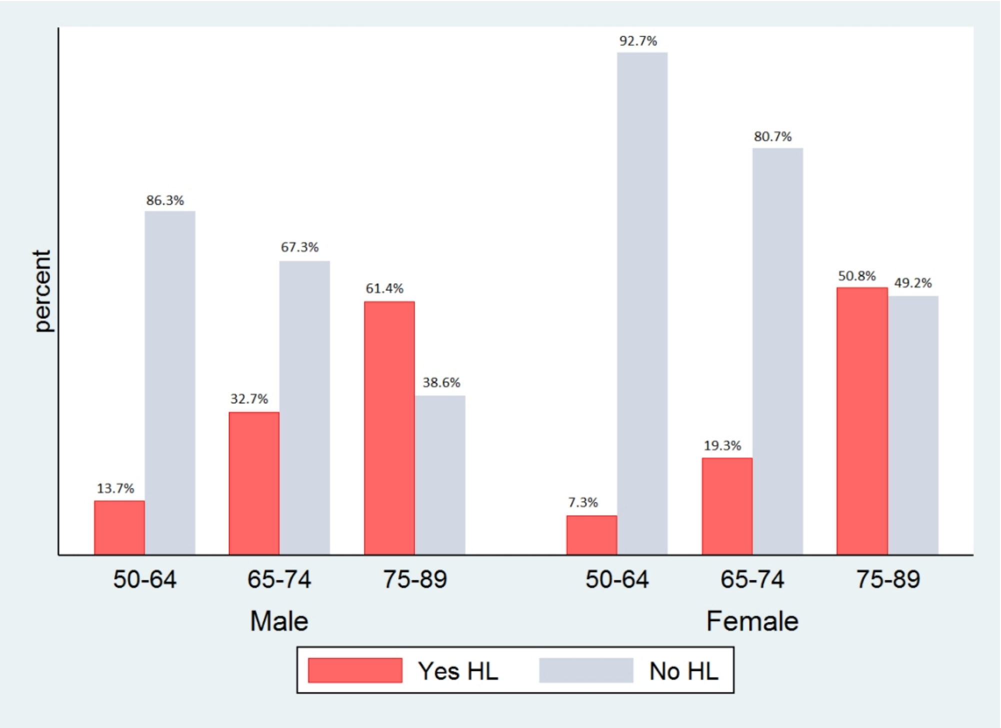

Figure 2.
Hearing loss by age group and gender (N=8,529 participants, aged 50-89, from the seventh wave of the English Longitudinal Study of Ageing (ELSA).
Hearing loss was defines as >35 dB HL at 3.0 kHz, in the better-hearing ear.

STROBE Statement—Checklist of items that should be included in reports of ***cross-sectional studies***

	Item No	Recommendation
**Title and abstract**	1	(*a*) Socioeconomic and lifestyle factors associated with hearing loss in older adults: A cross-sectional study of the English Longitudinal Study of Ageing (ELSA) **(Page 1)**
		(*b*) **Objectives**: Aims were (a) to examine whether socioeconomic position (SEP) is associated with hearing loss (HL) among older adults in England and (b) whether major modifiable lifestyle factors (high body mass index, physical inactivity, tobacco consumption and alcohol intake above the low risk level guidelines) are associated with HL after controlling for the effects of non-modifiable demographic factors and SEP.<br>**Setting:** We used data from the wave 7 of the English Longitudinal Study of Aging (ELSA), which is a longitudinal household survey dataset of a representative sample of people aged 50 and older in England.<br>**Participants**: The final analytical sample was n=8,529 participants, aged 50-89, who gave their consent to have their hearing acuity objectively measured by a screening audiometry device and did not have any ear infection.<br>**Primary and secondary outcome measures**: HL defined as >35 dB HL at 3.0 kHz, in the better-hearing ear. Those with HL were further subdivided into two categories depending on the number of tones heard at 3.0 kHz.<br>**Results**: HL was identified in 32.1% of men and 22.3% of women aged 50-89. Those in a lower SEP were up to two times more likely to have HL; the adjusted odds of HL were higher for those with no qualifications versus those with a degree/higher education, those in routine/manual occupations versus those in managerial/professional occupations, and those in the lowest versus the highest income and wealth quintiles. All regression models showed that socioeconomic and the modifiable lifestyle factors were strongly associated with HL after controlling for age and gender.<br>**Conclusions**: Our findings show that socioeconomic and lifestyle factors are associated with HL among older adults as strongly as core demographic risk factors, such as age and gender. Socioeconomic inequalities and modifiable lifestyle behaviours need to be targeted by health policy strategies, as an important step in designing interventions for individuals that face hearing health inequalities.<br>**(Page 2)**
**Introduction**		
Background/rationale	2	Prior research has established health disparities in a wide range of health conditions according to socioeconomic position (SEP). Furthermore, there is evidence that several modifiable lifestyle factors, such as smoking, alcohol consumption, high body mass index and physical inactivity are associated with bad hearing health. However, hearing health inequalities is an emerging research area and the existing evidence on the relationship of HL with SEP and modifiable lifestyle factors is limited. There is a

For peer review only - http://bmjopen.bmj.com/site/about/guidelines.xhtml

		major public health need to assess whether HL is associated with SEP and lifestyle factors because this understanding could inform recommendations for HL preventative strategies. These could include wider implementation of interventions to promote 'healthier lifestyles', or governmental policies for socioeconomic equity among older people in the community.<br>**(Page 4)**
Objectives	3	The aims of this study were (a) to examine whether SEP is associated with HL among older adults in England and (b) whether major modifiable lifestyle factors are associated with HL after controlling for the effects of non-modifiable demographic factors and SEP.<br>**(Page 5)**
**Methods**		
Study design	4	The present study used data from the English Longitudinal Study of Aging (ELSA). The ELSA is a longitudinal household survey dataset of a representative sample of people aged 50 and older in England. It is designed as a large-scale prospective cohort study, with repeat measures of core variables over numerous waves, in order to explore trajectories on the health, social, wellbeing and economic circumstances.<br>**(Page 5)**
Setting	5	The current sample contains data from up to eight waves of data collection covering a period of 15 years, with an ongoing two-year follow-up longitudinal design. Objective hearing health data was available only in wave 7, where information was collected from 9,666 participants, between June 2014 and May 2015.<br>**(Page 5)**
Participants	6	For the purposes of this study, the final analytical sample was n=8,529 participants, aged 50-89, who gave their consent to have their hearing acuity measured by a screening audiometry device and did not have any ear infection or a cochlear implant.<br>**(Page 5)**
Variables	7	**Outcomes**<br>*Hearing loss*<br>HL was defined as >35 dB HL at 3.0 kHz, in the better-hearing ear. Those with HL were further subdivided into two categories depending on the number of tones heard at 3.0 kHz. This is the level where intervention for HL has been shown as definitely beneficial. For that reason this categorisation has previously been used in the literature for the characterisation of those assessed by the same audiometric screening device (HearCheck™). Thus, we further explored potential differences in the association between SEP indicators and HL, according to the severity of HL, as measured by HearCheck™. The categorization of those with HL was as following:<br>(a) "*Moderate HL*": tones heard at 75 dB HL and 55 dB HL but not at 35 dB HL (the first 2 of the three tones at 3.0 kHz heard),<br>(b) "*Moderately severe or severe HL*": tone heard or not at 75 dB HL and tones not heard at 55 dB HL and 35 dB HL (0 or 1 of the three tones at 3.0 kHz heard).<br>The ordinal variable "hearing acuity" (in better ear) was consisted of the above two categories of HL and the category of "normal hearing", which was defined as having

For peer review only

heard all the three tones of the hearing screening test at 3.0 kHz.

Indicators of socioeconomic position

Education, occupation, income and wealth were the four selected indicators of SEP and information was collected in the seventh wave of ELSA, between June 2014 and May 2015. We considered five categories of the highest educational attainment: degree/higher education; A level (Level 3 of the National Qualifications Framework); O levels CSE (Certificate of Secondary Education); foreign/other; no qualifications. Tertiles of self-reported occupation were based on the National Statistics socio-economic classification (NS-SEC): managerial and professional; intermediate; routine and manual occupations). The relative financial position of the participants was captured by quintiles of the net household income (first quintile lowest; fifth quintile highest) that is summed across household members. In order to avoid the information bias that is related to the retirement status, we used quintiles of the total non-pension wealth that is reported at the household level (first quintile lowest; fifth quintile highest), which represents the sum of net financial wealth, net physical wealth and net housing wealth.

Covariates

Age, marital status, retirement status and non-medical determinants of health (body mass index, physical activity, tobacco and alcohol consumption) were assessed as covariates in the association between SEP indicators and HL.

Age was categorised into three groups (50-64, 65-74, 75-89), to allow for a comparison with Benova et al., who examined the association of socioeconomic position with hearing difficulty in ELSA wave 2.

Marital status was dichotomised into those that are currently married or not. Those who are currently married included the categories a) married, first and only marriage, b) in a registered Civil Partnership, c) remarried, in a second or later marriage. Those that categorised as not currently married included the categories a) single, that is never married and never registered in a marriage, b) separated, but still legally married, c) divorced, d) widowed.

Retirement status was dichotomised into those who were retired or not, according to the self-reported employment status.

Body Mass Index (BMI) measurements were grouped in four categories, according to WHO definitions: (a) underweight: BMI under 18.5, (b) normal: BMI 18.5 or over but less than 25, (c) overweight: BMI 25 or over but less than 30, and (d) obese: BMI 30 or over.

Tobacco consumption of any type of nicotine products was recoded into three categories: those that were current smokers, those that were former smokers and those that never smoked. Both current and former smokers answered the question of 'number of cigarettes smoked per day', to explore whether they were occasional or regular smokers.

Alcohol consumption was recorded using several continuous variables such as the number of days of alcohol consumption in the last seven days and the number of (a)

For peer review only - http://bmjopen.bmj.com/site/about/guidelines.xhtml

		measures of spirit, (b) glasses of wine and (c) pints of beer that the respondents had consumed during this period. We constructed a continuous variable to represent the sum of units of alcohol that the participants consumed in the last seven days, according to the Chief Medical Officer's Drinking Guidelines, that counts as 1 unit each measure of spirit and as 2 units each glass of wine of pint of beer. The constructed variable of units of alcohol during the last seven days was further dichotomised into those that consumed more than 14 units of alcohol the last seven days or not, in a separate variable.<br>Levels of physical activity were described by three ordinal variables that examined the frequency that the respondents do rigorous, moderate or mild sports or activities, with possible answers (a) more than once a week, (b) once a week, (c) one to three times a month and (d) hardly ever, or never.<br>**(Pages 6-9)**
Data sources/ measurement	8*	A handheld audiometric screening device (HearCheck™) was used for the objective measurement of hearing acuity. This is a portable and easy-to-use hearing screening test by Siemens, that tests for audibility of pure tone beeps, according to the number of tones that the respondent can hear for each sequence (at 1.0 kHz and 3.0 kHz), per each ear. The functional test sequence begins with a series of three sounds, that have decreasing volume at 1.0 kHz (55 dB HL, 34 dB HL, 20 dB HL) and afterwards another three sounds with decreasing volume at 3.0 kHz (75 dB HL, 55 dB HL, 35 dB HL).<br>**(Page 6)**
Bias	9	Prerequisites for the test were the device to make proper contact with the ear that is tested, hearing aid(s), glasses, earrings and hair bands to be removed to prevent from getting in the way of the hearing device and the room to be as quiet as possible. Participants indicated when they hear the sound by raising their finger. The total number of tones that the participants indicated they could hear in the sequence of sounds at 1.0 kHz and 3.0. kHz, per each ear, was recorded and the total tones heard in the better ear used for the categorization of those with HL.<br>In order to avoid the information bias that is related to the retirement status, we used quintiles of the total non-pension wealth that is reported at the household level (first quintile lowest; fifth quintile highest), which represents the sum of net financial wealth, net physical wealth and net housing wealth.<br>**(Page 6)**
Study size	10	Information was collected from 9,666 participants, between June 2014 and May 2015. For the purposes of this study, the final analytical sample was n=8,529 participants, aged 50-89, who gave their consent to have their hearing acuity measured by a screening audiometry device and did not have any ear infection or a cochlear implant. All the 8,529 individuals (of the 9,666 initial sample in ELSA wave 7), had usable objective hearing data, measured by a qualified nurse.<br>**(Page 5)**
Quantitative variables	11	Those with HL were further subdivided into two categories depending on the number of tones heard at 3.0 kHz. This is the level where intervention for HL has been shown

For peer review only

For peer review only - http://bmjopen.bmj.com/site/about/guidelines.xhtml

		as definitely beneficial. For that reason this categorisation has previously been used in the literature for the characterisation of those assessed by the same audiometric screening device (HearCheck™). Thus, we further explored potential differences in the association between SEP indicators and HL, according to the severity of HL, as measured by HearCheck™. The categorization of those with HL was as following:<br>(a) "*Moderate HL*": tones heard at 75 dB HL and 55 dB HL but not at 35 dB HL (the first 2 of the three tones at 3.0 kHz heard),<br>(b) "*Moderately severe or severe HL*": tone heard or not at 75 dB HL and tones not heard at 55 dB HL and 35 dB HL (0 or 1 of the three tones at 3.0 kHz heard).<br>The ordinal variable "hearing acuity" (in better ear) was consisted of the above two categories of HL and the category of "normal hearing", which was defined as having heard all the three tones of the hearing screening test at 3.0 kHz.<br>**(Page 7)**
Statistical methods	12	Categorical variables are presented as absolute (n) and relative (%) frequencies, while continuous variables are presented using their mean and standard deviation. The Kolmogorov-Smirnov test and normal plots were used to test the normality of the quantitative variable distributions. All the 8,529 individuals (of the 9,666 initial sample in ELSA wave 7), had usable objective hearing data, measured by a qualified nurse. In total, 257 participants refused to have the assessment (the 2.6% of the full cohort of 9,666 participants). As there was no pattern in the missing data regarding age, sex, education, occupation, income and wealth and due to low proportion of missingness (<5%), records with missing data were dropped from the analyses.<br>We fitted multiple logistic regression models to evaluate the odds of HL at various socioeconomic strata, controlling for gender, age and non-medical determinants of health (BMI, physical activity, tobacco and alcohol consumption). Additionally, we fitted four separate stepwise logistic regression models, to examine the association of HL with non-modifiable (age, gender: Step 1), partly modifiable (education, occupation, income, wealth: Step 2, respectively), and fully modifiable lifestyle risk factors (body mass index, physical activity, tobacco and alcohol consumption: Step 3). Age was entered into the multivariable logistic regression models as a continuous variable, to maximise power.<br>The variants of pseudo R squared statistics were based on the deviance of the models and used to express how much variance in the outcome is explained by the variables in each stepwise multiple logistic regression model. The variance inflation factor (VIF) was used as an indicator of multicollinearity and the Hosmer-Lemeshow test was used as a post estimation tool, which quantified the goodness-of-fit of the models. For all models, odds ratios, 95% confidence intervals, unadjusted and adjusted coefficients' beta values, pseudo R^2 and mean VIFs are presented. The two-tailed significance level was set ≤0.05. All data were analysed using Stata version 14.<br>**(Pages 9-10)**
		All the 8,529 individuals (of the 9,666 initial sample in ELSA wave 7), had usable objective hearing data, measured by a qualified nurse. In total, 257 participants

For peer review only

For peer review only - http://bmjopen.bmj.com/site/about/guidelines.xhtml

		refused to have the assessment (the 2.6% of the full cohort of 9,666 participants). As there was no pattern in the missing data regarding age, sex, education, occupation, income and wealth and due to low proportion of missingness (<5%), records with missing data were dropped from the analyses.<br>**(Page 9)**
**Results**		
Participants	13*	All the 8,529 individuals (of the 9,666 initial sample in ELSA wave 7), had usable objective hearing data and included in the study.
		In total, 257 participants refused to have the assessment (the 2.6% of the full cohort of 9,666 participants). As there was no pattern in the missing data regarding age, sex, education, occupation, income and wealth and due to low proportion of missingness (<5%), records with missing data were dropped from the analyses.<br>**(Page 9)**
Descriptive data	14*	The analyses were based on a representative cohort of 8,529 participants contained in ELSA, which is a rich resource of information on the dynamics of health, social, wellbeing and economic circumstances in the English population aged 50 and older<br>**(Page 5)**
		All the 8,529 individuals (of the 9,666 initial sample in ELSA wave 7), had usable objective hearing data and included in the study. In total, 257 participants refused to have the assessment (the 2.6% of the full cohort of 9,666 participants), but there was no pattern in the missing data regarding age, sex, education, occupation<br>**(Page 9)**
Outcome data	15*	Overall, 26.6% (2,266/8,529) of adults aged 50-89 had HL >35 dB HL at 3.0 kHz. The percentages were 32.1% (1,198/3,728, 95%CI 0.31 to 0.34) for men and 22.3% (1,068/4,801, 95%CI 0.21 to 0.23) for women, respectively.<br>**(Page 10)**
Main results	16	The proportion of men and women with HL >35 dB HL at 3.0kHz was 53.9 (1,158) and 46.2 (994), respectively. Men were 1.5 times more likely to have moderately severe or severe HL compared to women. One in three adults aged 65-75 had hearing loss and the percentage of HL in age band 75-89 was threefold larger than in age band 50-64, as one out of every two adults aged 75-89 had HL >35 dB HL at 3.0kHz.<br>The adjusted odds of HL were higher for those with no qualifications versus those with a degree/higher education (men: OR 1.87, 95% CI 1.47-2.38, women: OR 1.53, 95% CI 1.21-1.95), those in routine/manual occupations versus those in managerial/professional occupations (men: OR 1.92, 95% CI 1.43-2.63, women: OR 1.25, 95% CI 1.03-1.54), and those in the lowest versus the highest income and wealth quintiles (men: OR 1.62, 95% CI 1.08-2.44, women: OR 1.36, 95% CI 0.85-2.16 and men: OR 1.72, 95% CI 1.26-2.35, women: OR 1.88, 95% CI 1.37-2.58, respectively).<br>All regression models were statistically significant. Age and gender only explained about 15% of the variance in the likelihood of HL.<br>**(Pages 10-11)**
Other analyses	17	The addition of lifestyle factors attenuated significantly the association between the HL and SEP indicators and in total the addition of SEP and lifestyle factors in the regression models explained another 10 to 15% of the variance in the likelihood of

For peer review only

For peer review only - http://bmjopen.bmj.com/site/about/guidelines.xhtml

		HL. The total variance explained in the overall models containing demographic factors, SEP and lifestyle factors ranged between 25 and 27%. This finding suggests that SEP and lifestyle factors have an equal contribution to HL as age and gender. **(Page 12)**
**Discussion**		
Key results	18	This study found that variation in education, occupation, income and wealth, which are important determinants of health inequality, are associated with HL. SEP was strongly associated with the likelihood of HL in older adults, with the higher levels of education, income and wealth being less likely to be associated with HL, and the manual occupations increased the likelihood of HL. We also found that socioeconomic and several modifiable lifestyle factors (such as high body mass index, physical inactivity, tobacco consumption and alcohol intake above the low risk level guidelines) are associated with the likelihood of HL as strongly as well-established demographic factors such as age and gender HL. **(Page 12)**
Limitations	19	No causal or temporal relationships can be established between lifestyle factors and HL in this cross-sectional study. Unhealthy lifestyle behaviours could lead to HL in older people but is also possible that older people adopt less healthy lifestyles after HL. Second, all the analysed factors explained less than one third of the variance for the occurrence of HL suggesting that there are additional major factors associated with HL in older adults which have not been included in our analyses. Longitudinal analyses using a broader range of physical health, mental health and social care variables are highly recommended to obtain a comprehensive understanding of modifiable factors which contribute to HL among older adults in England. Third, the ELSA dataset did not include information concerning the occupational and social noise exposure, which has a damaging effect in hearing. We therefore were not able to examine the association of noise exposure with smoking in the relationship of SEP with HL, as in a previous study which found that the smoking habit in workers exposed to occupational noise greatly influenced HL. However, we examined the association of manual occupations with HL and its attenuation by modifiable determinants including smoking habit, which is of a higher prevalence among those that work in routine and manual occupations in England. **(Page 13)**
Interpretation	20	The findings show that HL is strongly associated with socioeconomic factors and modifiable lifestyle behaviours. These findings suggest that a large proportion of HL burden is potentially preventable and show that there is serious potential to reduce the prevalence and impacts of HL by understanding the impact of socioeconomic inequality in hearing health. Our findings are supportive of a new conceptualisation of HL which argues that HL is not necessarily an inevitable accompaniment of ageing, but also a potential preventable lifestyle disease, paving the way for the term *lifestyle-related hearing loss*, where *lifestyle* refers to *social practices and ways of living adopted by individuals that reflect personal, group, and socio-economic identities*, instead of the non-inclusive term "*age-related hearing loss*". **(Page 16)**
Generalisability	21	We used data from the wave 7 of the English Longitudinal Study of Aging (ELSA),

For peer review only

		which is a longitudinal household survey dataset of a representative sample of people aged 50 and older in England. However, we did not use in our analyses the non-response statistical weights for the refreshment sample members, who were selected from HSE 2011 and 2012, which may have reduced the generalizability of our findings. **(Page 14)**
**Other information**		
Funding	22	This research was funded by the NIHR Manchester Biomedical Research Centre (BRC). The views expressed are those of the authors and not necessarily those of the BRC, the NIHR or the Department of Health. **(Page 17)**

*Give information separately for exposed and unexposed groups.

Note: An Explanation and Elaboration article discusses each checklist item and gives methodological background and published examples of transparent reporting. The STROBE checklist is best used in conjunction with this article (freely available on the Web sites of PLoS Medicine at http://www.plosmedicine.org/, Annals of Internal Medicine at http://www.annals.org/, and Epidemiology at http://www.epidem.com/). Information on the STROBE Initiative is available at www.strobe-statement.org.

For peer review only

For peer review only - http://bmjopen.bmj.com/site/about/guidelines.xhtml

BMJ Open

Socioeconomic and Lifestyle Factors Associated with Hearing Loss in Older Adults: A Cross-sectional Study of the English Longitudinal Study of Ageing (ELSA)

Journal:	*BMJ Open*
Manuscript ID	bmjopen-2019-031030.R2
Article Type:	Research
Date Submitted by the Author:	15-Aug-2019

Complete List of Authors:	Tsimpida, Dialechti; University of Manchester, Centre for Primary Care and Health Services Research, Division of Population Health, School of Health Sciences, Faculty of Biology, Medicine and Health<br>Kontopantelis, Evangelos; University of Manchester, Division of Informatics, Imaging & Data Sciences, School of Health Sciences, Faculty of Biology, Medicine and Health, The University of Manchester<br>Ashcroft, Darren; University of Manchester, NIHR Greater Manchester Patient Safety Translational Research Centre, Division of Pharmacy and Optometry, School of Health Sciences, Faculty of Biology, Medicine and Health, The University of Manchester, Manchester, UK<br>Panagioti, Maria; University of Manchester, NIHR Greater Manchester Patient Safety Translational Research Centre, Division of Population Health, School of Health Sciences, Faculty of Biology, Medicine and Health, The University of Manchester, Manchester, UK

**Primary Subject Heading**:	Public health
Secondary Subject Heading:	Public health, Epidemiology
Keywords:	PUBLIC HEALTH, Hearing loss, socioeconomic position, hearing health inequalities, healthy ageing, social epidemiology

SCHOLARONE™
Manuscripts

TITLE PAGE

Socioeconomic and Lifestyle Factors Associated with Hearing Loss in Older Adults: A Cross-sectional Study of the English Longitudinal Study of Ageing (ELSA)

Dialechti Tsimpida,*
NIHR Doctoral Research Fellow, Centre for Primary Care and Health Services Research, Division of Population Health, School of Health Sciences, Faculty of Biology, Medicine and Health, The University of Manchester, Manchester, UK
** Corresponding author: Centre for Primary Care and Health Services Research,*
5th floor Williamson Building, The University of Manchester,
Oxford Road, M139PL, Manchester, UK
Tel.: +44 161 275 7006
E-mail address: dialechti.tsimpida@manchester.ac.uk

Professor Evangelos Kontopantelis,
Division of Informatics, Imaging & Data Sciences, School of Health Sciences, Faculty of Biology, Medicine and Health, The University of Manchester, Manchester, UK
E-mail address: e.kontopantelis@manchester.ac.uk

Professor Darren Ashcroft,
NIHR Greater Manchester Patient Safety Translational Research Centre, Division of Pharmacy and Optometry, School of Health Sciences, Faculty of Biology, Medicine and Health, The University of Manchester, Manchester, UK
E-mail address: Darren.Ashcroft@manchester.ac.uk

Dr Maria Panagioti,
Senior Lecturer, NIHR Greater Manchester Patient Safety Translational Research Centre, Division of Population Health, School of Health Sciences, Faculty of Biology, Medicine and Health, The University of Manchester, Manchester, UK
E-mail address: maria.panagioti@manchester.ac.uk

For peer review only

Keywords: Hearing loss; socioeconomic position; hearing health inequalities; healthy ageing; social epidemiology

Word count for the abstract; **297**

Manuscript word count; **4,044**

Number of references; **39**

For peer review only - http://bmjopen.bmj.com/site/about/guidelines.xhtml

ABSTRACT

Objectives: Aims were (a) to examine whether socioeconomic position (SEP) is associated with hearing loss (HL) among older adults in England and (b) whether major modifiable lifestyle factors (high body mass index, physical inactivity, tobacco consumption and alcohol intake above the low risk level guidelines) are associated with HL after controlling for non-modifiable demographic factors and SEP.

Setting: We used data from the wave 7 of the English Longitudinal Study of Ageing (ELSA), which is a longitudinal household survey dataset of a representative sample of people aged 50 and older.

Participants: The final analytical sample was 8,529 participants aged 50-89 that gave consent to have their hearing acuity objectively measured by a screening audiometry device and did not have any ear infection.

Primary and secondary outcome measures: HL defined as >35 dBHL at 3.0 kHz (better-hearing ear). Those with HL were further subdivided into two categories depending on the number of tones heard at 3.0kHz.

Results: HL was identified in 32.1% of men and 22.3% of women aged 50-89. Those in a lower SEP were up to two times more likely to have HL; the adjusted odds of HL were higher for those with no qualifications versus those with a degree/higher education, those in routine/manual occupations versus those in managerial/professional occupations, and those in the lowest versus the highest income and wealth quintiles. All regression models showed that socioeconomic and the modifiable lifestyle factors were strongly associated with HL after controlling for age and gender.

Conclusions: Socioeconomic and lifestyle factors are associated with HL among older adults as strongly as core demographic risk factors, such as age and gender. Socioeconomic inequalities and modifiable lifestyle behaviours need to be targeted by health policy strategies, as an important step in designing interventions for individuals that face hearing health inequalities.

For peer review only

For peer review only - http://bmjopen.bmj.com/site/about/guidelines.xhtml

Strengths and limitations of this study

- The first study that focuses on modifiable lifestyle factors (such as high body mass index, physical inactivity, tobacco consumption and alcohol intake above the low risk level guidelines) associated with HL among older adults in England.
- Examines four different SEP indicators to HL (education, occupation, income, wealth), instead of a proxy measure to reflect one's total SEP, capturing therefore most of the variation in socioeconomic stratification, to the objectively measured HL in older adults.
- The analyses were based on a representative cohort of 8,529 participants contained in ELSA, which is a rich resource of information on the dynamics of health, social, wellbeing and economic circumstances of the English population aged 50 and over.
- The ELSA dataset did not contain information concerning the occupational and social noise exposure, but we examined the association of manual occupations with HL and its attenuation by modifiable determinants including smoking habit, which is of a higher prevalence among those that work in routine and manual occupations in England.
- All the analysed factors explained less than one third of the variance for the prevalence of HL suggesting that there are additional major factors associated with HL in older adults which have not been included in our analyses.

For peer review only - http://bmjopen.bmj.com/site/about/guidelines.xhtml

INTRODUCTION

Hearing loss (HL) is a major global health challenge and the most prevalent sensory disorder. Approximately 15% of the global adult population has some degree of HL (of at least ≥25 dB HL in the better-hearing ear)[1] and almost 7% has disabling HL (defined as a hearing threshold ≥40 dB HL in the better ear)[2]. HL has negative physical, social, cognitive, economic and emotional consequences and is the fourth leading contributor to years lived with disability worldwide[2].

Previous studies have reported that HL increases with age[3], exposure to high occupational and social noise[4] and is more commonly in men[3]. There is growing evidence that there are a number of modifiable risk factors for HL[5,6] and, if eliminated, half cases of HL could be prevented [2]. Thus there is a high potential for reducing the burden of HL, if we understand the modifiable factors and the mechanisms that lead to hearing health inequalities, which -following the glossary for health inequalities[7]- could be defined as the *avoidable differences in people's hearing health across different social and/or population groups*.

Prior research has established health disparities in a wide range of health conditions according to socioeconomic position (SEP)[8]. Furthermore, there is evidence that several modifiable lifestyle factors, such as smoking[9], alcohol consumption[10], high body mass index and physical inactivity[11] are associated with hearing health. Of course, causal paths have not been established, and these associations may be confounded by deprivation or aspects of deprivation (e.g. type of occupation). Nevertheless, quantifying such associations is the first step in that direction; hearing health inequalities is an emerging research area and the existing evidence on the relationship of HL with SEP and modifiable lifestyle factors is scarce. There is a major public health need to assess whether HL is associated with SEP and lifestyle factors because this understanding could inform recommendations for HL preventative strategies. These could include wider implementation of interventions to promote 'healthier lifestyles', or governmental policies for socioeconomic equity among older people in the community.

The aims of this study were (a) to examine whether SEP is associated with HL among older adults in England and (b) whether major modifiable lifestyle factors are associated with HL after

For peer review only - http://bmjopen.bmj.com/site/about/guidelines.xhtml

controlling for non-modifiable demographic factors and SEP in the analyses. This study is the first that examines four different SEP indicators (education, occupation, income, wealth) in HL, encompassing thus aspects of the life-course socioeconomic stratification[12], to the objectively measured HL in older adults. In addition, this is the first study that explores how major lifestyle factors for general health outcomes in the English population aged 50 years old and above (such as smoking, high BMI, insufficient physical activity, tobacco consumption and alcohol intake above the low risk level guidelines)[13,14] account for the variance in HL.

For peer review only

METHODS

Study population

The present study used data from the English Longitudinal Study of Ageing (ELSA). The ELSA is a longitudinal household survey dataset of a representative sample of people aged 50 and older in England. It is designed as a large-scale prospective cohort study, with repeat measures of core variables over numerous waves, in order to explore trajectories on the health, social, wellbeing and economic circumstances.[15] The current sample contains data from up to eight waves of data collection covering a period of 15 years, with an ongoing two-year follow-up longitudinal design.[16]

Objective hearing health data was available only in wave 7, where information was collected from 9,666 participants, between June 2014 and May 2015. For the purposes of this study, the final analytical sample was n=8,529 participants, aged 50-89, that gave consent to have their hearing acuity measured by a screening audiometry device and did not have any ear infection or a cochlear implant.

Patient and Public Involvement

Patients were not involved in the conduct of the study.

For peer review only - http://bmjopen.bmj.com/site/about/guidelines.xhtml

Hearing test

A handheld audiometric screening device (HearCheck™)[17] was used for the objective measurement of hearing acuity. This is a portable and easy-to-use hearing screening test by Siemens, that tests for audibility of pure tone beeps, according to the number of tones that the respondent can hear for each sequence (at 1.0 kHz and 3.0 kHz), per each ear. The functional test sequence begins with a series of three sounds, that have decreasing volume at 1.0 kHz (55 dB HL, 34 dB HL, 20 dB HL) and afterwards another three sounds with decreasing volume at 3.0 kHz (75 dB HL, 55 dB HL, 35 dB HL). Prerequisites for the test were the device to make proper contact with the ear that is tested, hearing aid(s), glasses, earrings and hair bands to be removed to prevent from getting in the way of the hearing device and the room to be as quiet as possible. Participants indicated when they hear the sound by raising their finger. The total number of tones that the participants indicated they could hear in the sequence of sounds at 1.0 kHz and 3.0. kHz, per each ear, was recorded and the total tones heard in the better-hearing ear used for the categorization of those with HL.

Previous studies have assessed the accuracy of the Siemens HearCheck™ in detecting hearing loss and compared it with pure tone air conduction averages designated as gold standard values. Fellizan-Lopez *et al.* (2011) found that in cases of moderate or worse hearing loss, the HearCheck™ test fulfils all criteria of high sensitivity rate, high specificity rate and high positive predictive values to be considered an accurate tool to screen for hearing loss, without the need for soundproof audiometry booths[18].

Outcomes

Hearing loss

HL was defined as >35 dB HL at 3.0 kHz, in the better-hearing ear. Those with HL were further subdivided into two categories depending on the number of tones heard at 3.0 kHz. This is the level where intervention for HL has shown to be definitely beneficial.[19] For that reason this

For peer review only - http://bmjopen.bmj.com/site/about/guidelines.xhtml

categorisation has previously been used in the literature for the characterisation of those assessed by the same audiometric screening device (HearCheck™)[6]. Thus, we further explored potential differences in the association between SEP indicators and HL, according to the severity of HL, as measured by HearCheck™. The categorization of those with HL was as following:

(a) "*Moderate HL*": tones heard at 75 dB HL and 55 dB HL but not at 35 dB HL (the first 2 of the three tones at 3.0 kHz heard),

(b) "*Moderately severe or severe HL*": tone heard or not at 75 dB HL and tones not heard at 55 dB HL and 35 dB HL (0 or 1 of the three tones at 3.0 kHz heard).

The ordinal variable "hearing acuity" (in the better-hearing ear) was consisted of the above two categories of HL and the category of "normal hearing", which was defined as having heard all the three tones of the hearing screening test at 3.0 kHz.

Indicators of socioeconomic position

Education, occupation, income and wealth were the four selected indicators of SEP and information was collected in the seventh wave of ELSA, between June 2014 and May 2015. We considered five categories of the highest educational attainment: degree/higher education; A level (Level 3 of the National Qualifications Framework); O levels CSE (Certificate of Secondary Education); foreign/other; no qualifications. Tertiles of self-reported occupation were based on the National Statistics socio-economic classification (NS-SEC): managerial and professional; intermediate; routine and manual occupations). The relative financial position of the participants was captured by quintiles of the net household income (first quintile lowest; fifth quintile highest) that is summed across household members. In order to avoid the information bias that is related to the retirement status, we used quintiles of the total non-pension wealth that is reported at the household level (first quintile lowest; fifth quintile highest), which represents the sum of net financial wealth, net physical wealth and net housing wealth.

Covariates

For peer review only - http://bmjopen.bmj.com/site/about/guidelines.xhtml

Age, marital status, retirement status and non-medical determinants of health (body mass index, physical activity, tobacco and alcohol consumption) were assessed as covariates in the association between SEP indicators and HL[5].

Age was categorised into three groups (50-64, 65-74, 75-89), to allow for a comparison with Benova et al.,[20] who examined the association of socioeconomic position with self-reported hearing difficulty in ELSA wave 2.

Marital status was dichotomised into those that are currently married or not. Those who are currently married included the categories a) married, first and only marriage, b) in a registered Civil Partnership, c) remarried, in a second or later marriage. Those that categorised as not currently married included the categories a) single, that is never married and never registered in a marriage, b) separated, but still legally married, c) divorced, d) widowed.

Retirement status was dichotomised into those who were retired or not, according to the self-reported employment status.

Body Mass Index (BMI) measurements were grouped in four categories, according to WHO definitions[21]: (a) underweight: BMI under 18.5, (b) normal: BMI 18.5 or over but less than 25, (c) overweight: BMI 25 or over but less than 30, and (d) obese: BMI 30 or over.

Tobacco consumption of any type of nicotine products was recoded into three categories: those that were current smokers, those that were former smokers and those that never smoked. Both current and former smokers answered the question of 'number of cigarettes smoked per day', to explore whether they were occasional or regular smokers.

Alcohol consumption was recorded using several continuous variables such as the number of days of alcohol consumption in the last seven days and the number of (a) measures of spirit, (b) glasses of wine and (c) pints of beer that the respondents had consumed during this period. We constructed a continuous variable to represent the sum of units of alcohol that the participants consumed in the last seven days, according to the Chief Medical Officer's Drinking Guidelines[22], that counts as 1 unit each measure of spirit and as 2 units each glass of wine of pint of beer. The constructed variable of units of alcohol during the last seven days was further dichotomised into those that consumed more than 14 units of alcohol the last seven days or not, in a separate variable.

For peer review only - http://bmjopen.bmj.com/site/about/guidelines.xhtml

Levels of physical activity were described by three ordinal variables that examined the frequency that the respondents do rigorous, moderate or mild sports or activities, with possible answers (a) more than once a week, (b) once a week, (c) one to three times a month and (d) hardly ever, or never.

Statistical analysis

Categorical variables are presented as absolute (n) and relative (%) frequencies, while continuous variables are presented using their mean and standard deviation. The Kolmogorov-Smirnov test and normal plots were used to test the normality of the quantitative variable distributions. All the 8,529 individuals (of the 9,666 initial sample in ELSA wave 7), had usable objective hearing data, measured by a qualified nurse. In total, 257 participants refused to have the assessment (the 2.6% of the full cohort of 9,666 participants). As there was no pattern in the missing data regarding age, sex, education, occupation, income and wealth and due to low proportion of missingness (<5%), records with missing data were dropped from the analyses.

We fitted multiple logistic regression models to evaluate the odds of HL at various socioeconomic strata, controlling for gender, age and non-medical determinants of health (BMI, physical activity, tobacco and alcohol consumption). Additionally, we fitted four separate stepwise logistic regression models, to examine the association of HL with non-modifiable (age, gender: Step 1), partly modifiable (education, occupation, income, wealth: Step 2, respectively), and fully modifiable lifestyle risk factors (body mass index, physical activity, tobacco and alcohol consumption: Step 3). Age was entered into the multivariable logistic regression models as a continuous variable, to maximise power.

The variants of pseudo R squared statistics were based on the deviance of the models and used to express how much variance in the outcome is explained by the variables in each stepwise multiple logistic regression model. The variance inflation factor (VIF) was used as an indicator of multicollinearity and the Hosmer-Lemeshow test was used as a post estimation tool, which quantified the goodness-of-fit of the models. For all models, odds ratios, 95% confidence intervals, unadjusted and adjusted coefficients' beta values, pseudo R^2 and mean VIFs are

For peer review only - http://bmjopen.bmj.com/site/about/guidelines.xhtml

presented. The two-tailed significance level was set ≤0.05. All data were analysed using Stata version 14 (StataCorp, 2015)[23].

RESULTS

Socio-demographic characteristics

Overall, 26.6% (2,266/8,529) of adults aged 50-89 had HL >35 dB HL at 3.0 kHz. The percentages were 32.1% (1,198/3,728, 95%CI 0.31 to 0.34) for men and 22.3% (1,068/4,801, 95%CI 0.21 to 0.23) for women, respectively. Table 1 shows the distribution of socio-demographic characteristics of the sample (n=8,529, aged 50-89) according to hearing acuity. The proportion of men and women with HL >35 dB HL at 3.0 kHz was 52.8 (1,198)and 47.2 (1,068)), respectively. However, men were 1.5 times more likely to have moderately severe or severe HL compared to women. One in three adults aged 65-75 had hearing loss and the percentage of HL in age band 75-89 was threefold larger than in age band 50-64, as one out of every two adults aged 75-89 had HL >35 dB HL at 3.0kHz.

Table 1

Lifestyle factors

Lifestyle factors of the participants are presented in Table 2. Over half of the participants were current or former smokers. In addition, patterns of high levels of alcohol consumption among all participants were revealed, with average consumption of more than 14 units of alcohol in the last seven days for two out of three participants (5,223/8,528, 95%CI 0.60 to 0.61). Nearly one out of every three of those drinking above the low risk level guidelines[22] (1,457/5.223, 95%CI 0.27 to 0.29) had HL >35 dB HL at 3.0 kHz.

For peer review only - http://bmjopen.bmj.com/site/about/guidelines.xhtml

Three out of four of those with HL >35 dB HL at 3.0 kHz were overweight or obese. Furthermore, those with HL >35 dB HL at 3.0 kHz were twice as likely to hardly ever or never engage in moderate or mild sports activities compared to hearing participants.

Table 2

Hearing Loss

Table 3 and Figure 1 show the results of multiple logistic regression analysis with HL >35 dB HL at 3.0 kHz as the dependent variable and SEP indicators as the independent variables, per each gender. The adjusted odds of HL were higher for those with no qualifications versus those with a degree/higher education (men: OR 1.87, 95% CI 1.47-2.38, women: OR 1.53, 95% CI 1.21-1.95), those in routine/manual occupations versus those in managerial/professional occupations (men: OR 1.92, 95% CI 1.43-2.63, women: OR 1.25, 95% CI 1.03-1.54), and those in the lowest versus the highest income and wealth quintiles (men: OR 1.62, 95% CI 1.08-2.44, women: OR 1.36, 95% CI 0.85-2.16 and men: OR 1.72, 95% CI 1.26-2.35, women: OR 1.88, 95% CI 1.37-2.58, respectively).

Table 3 & Figure 1

Table 4 shows the summary of stepwise logistic regression analysis for variables predicting HL >35 dB HL at 3.0 kHz. All regression models were statistically significant. Age and gender only explained about 15% of the variance in the likelihood of HL. The addition of lifestyle factors attenuated significantly the association between the HL and SEP indicators and in total the addition of SEP and lifestyle factors in the regression models explained another 10 to 15% of the variance in the likelihood of HL. The total variance explained in the overall models containing demographic factors, SEP and lifestyle factors ranged between 25 and 27%. This finding suggests that SEP and lifestyle factors have an equal contribution to HL as age and gender.

For peer review only - http://bmjopen.bmj.com/site/about/guidelines.xhtml

The differences in hearing loss prevalence between males and females were observed across all age bands investigated. However, we noticed that the rate of deterioration of hearing acuity as age increases was similar between each age band and nearly to 60% in both genders (Figure 2). The difference in prevalence begins at the age band “50-64”, where men were twice as likely to have HL.

Table 4

DISCUSSION

Summary of main findings

In this study we examined whether SEP and modifiable lifestyle factors are associated with HL among older adults in England. We found that variation in education, occupation, income and wealth, which are important determinants of health inequality, are associated with HL. SEP was strongly associated with the likelihood of HL in older adults, with the higher levels of education, income and wealth being less likely to be associated with HL, and the manual occupations increased the likelihood of HL. We also found that socioeconomic and several modifiable lifestyle factors (such as high body mass index, physical inactivity, tobacco consumption and alcohol intake above the low risk level guidelines[22]) are associated with the likelihood of HL as strongly as well-established demographic factors such as age and gender HL. These findings suggest that a large proportion of HL burden is potentially preventable and support the proposition of Scholes et al.[6], that there is serious potential to reduce the prevalence and impacts of HL by understanding the impact of socioeconomic inequality in hearing health. Thus, the incidence and severity of HL in England could be significantly reduced by governmental policies to mitigate socioeconomic disparities and public health interventions to promote healthier lifestyles in middle-aged and older adults in England. The occurrence of objective hearing data eliminated the different types of bias that occur in self-reporting hearing difficulties[24], strengthening the accuracy of findings.

For peer review only - http://bmjopen.bmj.com/site/about/guidelines.xhtml

Strengths and limitations

The main strength of our study was that is the first to examine the association of four separate SEP indicators with HL among older adults in England, instead of a proxy measure to reflect one's total SEP, capturing therefore most of the variation in socioeconomic stratification[12] and also the role of modifiable lifestyle risk factors in these associations. Another strength is that the analyses were based on a representative cohort of 8,529 participants contained in ELSA, which is a rich resource of information on the dynamics of health, social, wellbeing and economic circumstances in the English population aged 50 and older[16].

However there are also important limitations. First, no causal or temporal relationships can be established between lifestyle factors and HL in this cross-sectional study. Unhealthy lifestyle behaviours could lead to HL in older people but is also possible that older people adopt less healthy lifestyles after HL. Second, all the analysed factors explained less than one third of the variance for the prevalence of HL suggesting that there are additional major factors associated with HL in older adults which have not been included in our analyses. Longitudinal analyses using a broader range of physical health, mental health and social care variables are highly recommended to obtain a comprehensive understanding of modifiable factors which contribute to HL among older adults in England. Third, the ELSA dataset did not include information concerning the occupational and social noise exposure, which has a damaging effect in hearing[4]. We therefore were not able to examine the association of noise exposure with smoking in the relationship of SEP with HL, as in a previous study which found that the smoking habit in workers exposed to occupational noise greatly influenced HL[25]. However, we examined the association of manual occupations with HL and its attenuation by modifiable determinants including smoking habit, which is of a higher prevalence among those that work in routine and manual occupations in England[13]. Finally, we did not run weighted analyses which may have reduced the generalizability of our findings, as the ELSA sample members at Wave 7 could be healthier on average than the population, potentially resulting in an underestimation of relationships.

For peer review only - http://bmjopen.bmj.com/site/about/guidelines.xhtml

Research and policy implications

A number of previous studies have reported that the odds of HL in older adults were significantly increased for those with lower educational attainment [6,10,26,27], and those in manual versus non-manual occupations[28,29,30,31]. Besides, income is a correlate of HL, with the prevalence of untreated HL being higher among low-income older adults in the United States[31]. In our study, those in the lowest quintile of net household income had disproportionally higher percentages of moderate HL compared to moderately severe or severe HL, but this pattern was not found in the quintiles of wealth, as expected. This may indicate a possible delay in diagnosis of hearing problems among those in lower SEP due to financial barriers in access to health services[32], which needs further exploration, as HL is highly undiagnosed and untreated among older adults in England[20].

International studies have also shown that tobacco consumption, high body mass and high fat and high calorie food consumption can have an adverse impact on hearing 11[,33,34,]35 On the other hand, a higher level of physical activity is related with a lower risk of HL[34]. In our study, two out of three participants were drinking more than the low risk level of the 14 units of alcohol a week[22]. We considered therefore that alcohol consumption above the low risk level guidelines may play an important role in the association between SEP and HL among the English population and thus we included this variable in the regression models, which has not been previously examined in the literature for the English population. Our findings showed that drinking above the low risk level guidelines increased the likelihood of HL. This finding is in line with Chief Medical Officer's Drinking Guidelines[22,] which suggest that *it is safest not to drink regularly more than 14 units per week, to keep health risks from drinking alcohol to a low level*.

The associations between indicators of lower socioeconomic position and hearing loss may be markers of less healthy lifestyle[5], which may explain the link between HL and socioeconomic and lifestyle factors investigated. Cruickshanks et al, (2015) [36] did not find significant associations between hearing impairment and body mass index, smoking, and alcohol in multivariable analyses using a younger population-based sample (aged 18 to 74 years) of Hispanics/Latinos. Hence, it is likely that hearing loss in older population (e.g. 50 years and above) is associated with

For peer review only - http://bmjopen.bmj.com/site/about/guidelines.xhtml

different risk factors or combinations of socioeconomic and lifestyle risk factors across the life-course.

The higher prevalence of HL among men aged 50 and above compared to women has also been reported in other studies[3,6]. However, we observed that the rate of deterioration of hearing acuity as age increases was similar between each age band and nearly to 60% in both genders. The difference in prevalence begins at the age band “50-64”, where men were twice as likely to have HL. Thus, the differences in modifiable lifestyle factors that were revealed in the stepwise regression models may finally explain why the male sex is often cited as consistent risk factor for hearing loss[35,36,37], leading to the exploration of modifiable determinants that are common in both genders[5] and paving the way for interventions to improve the population’s hearing health.

In terms of policy, generating evidence concerning the critical variables associated with HL is an important step in designing targeted services and interventions for individuals that face hearing health inequalities, and especially for those in the lowest SEP groups, where the burden of HL falls highest. This is of major importance for the population in England, as sensor diseases are the first leading cause of morbidity among adults 70 years and older and the second leading cause among adults 50-69 years[13]. Our findings support the view that HL is a non-communicable disease[38] which can be prevented or ameliorated by governmental policies to mitigate socioeconomic disparities and public health interventions to promote healthier lifestyles in middle-aged and older adults in England.

Conclusion

The main finding of our study is that HL is strongly associated with socioeconomic factors and modifiable lifestyle behaviours. Our findings are supportive of a new conceptualisation of HL which argues that HL is not necessarily an inevitable accompaniment of ageing, but also a potential preventable lifestyle disease, paving the way for the term *lifestyle-related hearing loss*, where *lifestyle* refers to *social practices and ways of living adopted by individuals that reflect personal, group, and socio-economic identities*[39], instead of the non-inclusive term “*age-related hearing loss*”. Future research in hearing health inequalities should investigate the role of the

For peer review only - http://bmjopen.bmj.com/site/about/guidelines.xhtml

prolonged exposure to these modifiable lifestyle behaviours in the development of HL and the role of other comorbid chronic diseases in the elderly.

For peer review only

For peer review only - http://bmjopen.bmj.com/site/about/guidelines.xhtml

Contributors DT, EK, DA and MP were responsible for developing the design of the study. DT was responsible for conducting the analyses, interpreting the results and drafting the manuscript. DT, EK, DA and MP critically revised the manuscript. All authors have read and approved the final manuscript.

Acknowledgment DT would like to acknowledge the contribution of Dr Piers Dawes and Prof Neil Pendleton, who had provided assistance in obtaining funding and were the former supervisors for her NIHR Manchester Biomedical Research Centre PhD Studentship, but did not fulfil the criteria set out in the Authorship Guidelines of The University of Manchester to be listed as authors.

Funding This research was funded by the NIHR Manchester Biomedical Research Centre (BRC). The views expressed are those of the authors and not necessarily those of the BRC, the NIHR or the Department of Health.

Competing interests None declared

Patient consent Not required

Ethics approval Not required. The study used data from the English Longitudinal Study of Ageing (ELSA).

Provenance and peer review Non commissioned; externally peer reviewed

Data sharing statement The English Longitudinal Study of Ageing dataset is available via the UK Data Service (http://www.ukdataservice.ac.uk). Statistical code is available from the corresponding author at dialechti.tsimpida@manchester.ac.uk.

Open Access This is an Open Access article distributed in accordance with the Creative Commons Attribution Non Commercial (CC BY-NC 4.0) license, which permits others to distribute, remix, adapt, build upon this work non-commercially, and licence their derivative works on different terms, provided the original work is properly cited and the use is non-commercial. See: http://creativecommons.org/licenses/by-nc/4.0/

© Article author(s) (or their employer(s) unless otherwise stated in the text of the article) 2019. All rights reserved. No commercial use is permitted unless otherwise expressly granted.

For peer review only - http://bmjopen.bmj.com/site/about/guidelines.xhtml

Table 1

Participants socio-demographic characteristics (N=8,529, aged 50-89)

	Hearing acuity % (N) in the better-hearing ear			
**Variable**	**Normal Hearing**	**HL >35 dB HL at 3.0kHz**	**Moderate HL***	**Moderately severe or severe HL****
**Gender**				
Male	40.4 (2,530)	52.8 (1,198)	49.5 (741)	59.5 (457)
Female	59.6 (3,733)	47.2 (1,068)	50.5 (757)	40.5 (311)
**Age**[a]	64.3 (9.29)	69.7 (19.19)	70.0 (15.85)	69.1 (24.41)
**Age group**				
50-64	51.3 (3,135)	16.2 (349)	19.3 (280)	9.8 (69)
65-74	34.5 5 (2,108)	33.6 (722)	36.9 (535)	26.7 (187)
75-89	14.2 (868)	50.2 (1,081)	43.8 (636)	63.5 (445)
**Currently married**				
No	31.2 (1,908)	38.4 (826)	37.5 (544)	40.2 (282)
Yes	68.8 (4,202)	61.6 (1,326)	62.5 (907)	59.8 (701)
**Retirement status**				
Retired	52.4 (3,205)	78.3 (1,685)	76.6 (1,112)	81.3 (573)
Not retired	47.6 (2,905)	21.7 (467)	23.4 (339)	18.3 (128)
**Education**				
Degree/Higher Education	33.7 (1,996)	26.4 (562)	28.1 (404)	22.9 (158)
A level	10.0 (596)	6.4 (137)	7.0 (100)	5.4 (37)
O level/CSE grade	24.4 (1,448)	22.3 (473)	22.4 (321)	22.0 (152)
Foreign/Other	13.5 (798)	11.9 (252)	11.9 (171)	11.7 (81)
No qualifications	18.4 (1,090)	33.0 (701)	30.6 (439)	38.0 (262)
**Occupation based National Statistics Socio-economic Classification (NS-SEC)**				
Managerial and professional occupations	23.4 (1,158)	21.5 (423)	21.6 (285)	21.2 (138)
Intermediate occupations (non-manual)	43.4 (2,149)	33.8 (665)	36.2 (477)	28.9 (188)
Routine and manual occupations	33.2 (1,644)	44.7 (1,643)	42.2 (1,318)	49.9 (325)

(Continued)

For peer review only - http://bmjopen.bmj.com/site/about/guidelines.xhtml

Table 1 (Continued)
Participants socio-demographic characteristics (N=8,529, aged 50-89)

	Hearing acuity % (N) in the better-hearing ear			
**Variable**	**Normal Hearing**	**HL >35 dB HL at 3.0kHz**	**Moderate HL***	**Moderately severe or severe HL****
**Net Household Income**				
First quintile (lowest)	17.0 (872)	21.3 (421)	19.7 (262)	24.8 (159)
Second quintile	18.7 (959)	24.8 (489)	24.7 (329)	24.9 (160)
Third quintile	20.1 (1,034)	23.0 (453)	22.3 (297)	24.3 (156)
Fourth quintile	22.5 (1,154)	18.6 (367)	19.9 (265)	15.9 (102)
Fifth quintile (highest)	21.7 (1,112)	12.3 (243)	13.4 (178)	10.1 (65)
**Net Financial Wealth**				
First quintile (lowest)	15.5 (794)	14.7 (290)	14.9 (199)	14.2 (91)
Second quintile	17.1 (879)	24.1 (475)	22.1 (294)	28.2 (181)
Third quintile	19.6 (1,006)	23.6 (466)	23.4 (311)	24.1 (155)
Fourth quintile	23.5 (1,204)	20.3 (400)	21.3 (284)	18.1 (116)
Fifth quintile (highest)	24.3 (1,248)	17.3 (342)	18.3 (243)	15.4 (99)

Values are expressed as column % (N) unless otherwise is indicated.

[a]Mean (Standard deviation)

*Moderate hearing loss: tones heard at 75 dB HL and 55 dB HL but not at 35 dB HL (the first **2 of the three tones at 3.0 kHz** heard)
Moderately severe or severe hearing loss: tone heard or not at 75 dB HL and tones not heard at 55 dB HL and 35 dB HL (0 or 1 of the three tones at 3.0 kHz** heard).

For peer review only - http://bmjopen.bmj.com/site/about/guidelines.xhtml

Table 2
Participants' lifestyle factors (N=8,529, aged 50-89)

Variable	Normal Hearing (Hearing acuity % (N) in the better-hearing ear)	HL >35 dB HL at 3.0kHz	Moderate HL*	Moderately severe or severe HL**
**Tobacco consumption (any type of nicotine products)**				
Current	11.7 (712)	10.0 (215)	9.6 (139)	10.8 (76)
Former	49.0 (2,996)	56.7 (1,219)	55.8 (810)	58.4 (409)
Number of cigarettes smoked per day [a]	12.79 (14)	12.79 (13)	12.69 (13)	11.90 (12)
Never	39.3 (2,403)	33.3 (718)	34.6 (502)	30.8 (216)
**Alcohol consumption (in the last 7 days)**				
Number of days of alcohol consumption [b]	3 (3)	3 (4)	3 (4)	3 (4)
Number of measures of spirit [a]	2.1 (2)	2.3 (3)	2.2 (3)	2.6 (3)
Number of glasses of wine [a]	4.3 (6)	3.6 (5)	3.9 (6)	3.1 (4)
Number of pints of beer [a]	2.1 (2)	2.3 (3)	2.3 (3)	2.4 (3)
Total units of alcohol in the last 7 days [a]	15.0 (18)	14.2 (19)	14.5 (21)	13.5 (17)
Consumption of more than 14 units	61.6 (3,766)	67.7 (1,457)	67.3 (977)	68.5 (480)
**BMI Classification**				
Underweight	3.4 (160)	5.0 (92)	4.9 (60)	5.3 (32)
Normal	26.9 (1,255))	20.6 (376)	19.6 (239)	22.7 (137)
Overweight	40.0 (1,869)	42.8 (780)	41.4 (506)	45.4 (274)
Obese	29.7 (1,390)	31.6 (576)	34.1 (416)	26.6 (160))
**Physical Activity**				
Frequency does rigorous sports or activities				
More than once a week	23.0 (1,407)	14.3 (307)	16.1 (233)	10.6 (74)
Once a week	10.3 (626)	7.0 (151)	7.9 (115)	5.1 (36)
One to three times a month	10.1 (617)	7.1 (153)	7.6 (111)	6.0 (42)
Hardly ever, or never	56.6 (3,459)	71.6 (1,541)	68.4 (992)	78.3 (549)

(Continued)

Table 2 (Continued)

Participants' lifestyle factors (N=8,529, aged 50-89)

Variable	Hearing acuity % (N) in the better-hearing ear			
	Normal Hearing	HL >35 dB HL at 3.0kHz	Moderate HL*	Moderately severe or severe HL**
**Physical Activity** (continued)				
Frequency does moderate sports or activities				
More than once a week	68.4 (4,180)	51.3 (1,104)	53.7 (780)	46.2 (324)
Once a week	12.6 (771)	13.6 (292)	14.1 (204)	12.6 (88)
One to three times a month	5.9 (360)	7.8 (169)	7.6 (110)	8.4 (59)
Hardly ever, or never	13.1 (799)	27.3 (587)	24.6 (357)	32.8 (230)
Frequency does mild sports or activities				
More than once a week	83.9	73.7	76.0 (1,103)	68.9 (483)
Once a week	8.2	10.1	9.8 (142)	10.5 (74)
One to three times a month	2.3	3.5	3.3 (48)	4.0 (28)
Hardly ever, or never	5.6	12.7	10.9 (158)	16.6 (116)

Values are expressed as column % (N) unless otherwise is indicated.

[a]Mean (Standard deviation)

[b]Median (Range)

*Moderate hearing loss: tones heard at 75 dB HL and 55 dB HL but not at 35 dB HL (the first **2 of the three tones at 3.0 kHz** heard)
Moderately severe or severe hearing loss: tone heard or not at 75 dB HL and tones not heard at 55 dB HL and 35 dB HL (0 or 1 of the three tones at 3.0 kHz** heard).

For peer review only - http://bmjopen.bmj.com/site/about/guidelines.xhtml

Table 3.
Multiple logistic regression analysis of N=8,529, aged 50-89 with **HL >35 dB HL at 3.0kHz** in better –hearing ear as dependent variable and **SEP indicators** as independent variables

	**Unadjusted OR (95% CI)***		**Adjusted OR (95% CI)****	
	Men	Women	Men	Women
**Education**				
No qualifications	2.39 (1.96-2.90)	2.67 (2.20-3.24)	1.87 (1.47-2.38)	1.53 (1.21-1.95)
Foreign/Other	1.06 (0.83-1.36)	1.37 (1.07-1.74)	1.46 (1.09-1.94)	0.99 (0.74-1.32)
O level/CSE grade	1.56 (1.29-1.89)	1.00 (0.80-1.25)	1.42 (1.13-1.79)	0.94 (0.73-1.22)
A level	1.01 (0.77-1.32)	0.69 (0.50-0.97)	1.08 (0.78-1.51)	0.82 (0.56-1.21)
Degree/Higher Education (reference)				
**Occupation based National Statistics socio-economic classification (NS-SEC)**				
Routine and manual occupations	1.69 (1.39-2.08)	1.35 (1.15-1.59)	1.92 (1.43-2.63)	1.25 (1.03-1.54)
Intermediate occupations (non-manual)	1.47 (1.23-1.75)	1.54 (1.19-1.96)	1.61 (1.25-2.08)	1.35 (1.01-1.85)
Managerial and professional occupations (reference)				
**Net Household Income**				
First quintile (lowest)	1.94 (1.50-2.52)	3.04 (2.31-3.99)	1.62 (1.08-2.44)	1.36 (0.85-2.16)
Second quintile	2.12 (1.67-2.70)	3.00 (2.28-3.93)	1.31 (0.93-1.85)	1.40 (0.89-2.18)
Third quintile	1.98 (1.56-2.51)	2.31 (1.75-3.05)	1.40 (1.01-1.94)	1.08 (0.69-1.67)
Fourth quintile	1.38 (1.08-1.74)	1.65 (1.23-2.20)	1.09 (0.80-1.49)	1.08 (0.70-1.66)
Fifth quintile (highest) (reference)				
**Net Financial Wealth**				
First quintile (lowest)	1.11 (0.86-1.45)	1.79 (1.38-2.33)	1.72 (1.26-2.35)	1.88 (1.37-2.58)
Second quintile	1.92 (1.52-2.42)	2.39 (1.88-3.04)	1.66 (1.26-2.18)	1.33 (1.00-1.77)
Third quintile	1.63 (1.30-2.04)	1.95 (1.53-2.50)	1.45 (1.12-1.88)	1.41 (1.06-1.88)
Fourth quintile	1.06 (0.85-1.32)	1.48 (1.15-1.91)	0.96 (0.75-1.24)	1.26 (0.94-1.68)
Fifth quintile (highest) (reference)				

*Unadjusted odds ratio (OR) ** Odds Ratio adjusted for age, marital status, retirement status, body mass index, tobacco consumption, alcohol consumption and physical activity.

For peer review only

For peer review only - http://bmjopen.bmj.com/site/about/guidelines.xhtml

Table 4.
Summary of stepwise logistic regression coefficients for variables predicting **HL >35 dB HL at 3.0kHz in the better-hearing ear** (N=8,529, aged 50-89), according to different SEP indicators (education, occupation, income, wealth).

	Model A			Model B			Model C			Model D		
**Step/Predictor**	**Step 1**	**Step 2a**	**Step 3**	**Step 1**	**Step 2b**	**Step 3**	**Step 1**	**Step 2c**	**Step 3**	**Step 1**	**Step 2d**	**Step 3**
**1 Non-modifiable**	(Education)			(Occupation)			(Income)			(Wealth)		
Gender (female)	-.62***	-.59***	-.72***	-.62***	-.64***	-.68***	-.62***	-.69***	-.70***	-.62***	-.69***	-.62***
Age	.12***	.11***	.10***	.12***	.13***	.11***	.12***	.11***	.11***	.12***	.11***	.12***
**2 Partly modifiable**												
2a Education		-.15***	-.11***		-	-		-	-		-	-
2b Occupation (manual)			-		.26***	.20***		-	-		-	-
2c Net Household Income			-		-	-		-.14***	-.09***		-	-
2d Net Financial Wealth			-		-	-		-	-		-.17***	-.11***
**3 Modifiable**												
Smoking (current/former)			.10*			.09			.10*			.09**
Alcohol consumption (> 14 units per week)			.24***			.19***			.17***			.18**
Body mass index (<25)			-.05*			-.06			-.03			-.04
Physical Activity (*rigorous* sports or activities, once or more/week)			-.14***			-.16***			-.12***			-.13***
Physical Activity (*moderate* sports or activities, once or more/week)			-.24***			-.24***			-.24***			-.24***
Physical Activity (*mild* sports or activities, once or more/week)			-.17***			-.15***			-.15***			-.14***
Pseudo R^2	.15	.18	.28	.15	.19	.26	.17	.18	.29	.17	.18	.27
Δ Pseudo R^2	-	.03	.10	-	.04	.07	-	.01	.11	-	.01	.09
Mean VIF	-	-	1.16	-	-	1.20	-	-	1.24	-	-	1.15

$^*p < .05$. $^{**}p < .01$ $^{***}p < .001$

For peer review only

For peer review only - http://bmjopen.bmj.com/site/about/guidelines.xhtml

Figure 1.

Associations between socioeconomic position and hearing loss in middle aged and older adults (N=8,529, aged 50-89). Indicators of SEP were categories of the highest educational attainment (degree/higher education as a reference), tertiles of self-reported occupation based on the National Statistics Socio-economic Classification (NS-SEC) (managerial and professional as reference), quintiles of the net household income (first quintile lowest; fifth quintile highest) and quintiles of the total non-pension wealth that is reported at the household level (first quintile lowest; fifth quintile highest). Lines represent OR (outcome=hearing loss) and its 95% CI. Model A (rhombus): unadjusted. Model B (circles): adjusted for age, marital status, retirement status, body mass index, tobacco consumption, alcohol consumption and physical activity.

Figure 2.

Hearing loss by age group and gender* (N=8,529 participants, aged 50-89, from the seventh wave of the English Longitudinal Study of Ageing (ELSA).

Hearing loss was defined as >35 dB HL at 3.0 kHz, in the better-hearing ear.

*Prevalence estimates for males (N=3,728) and females (N=4,801).

For peer review only - http://bmjopen.bmj.com/site/about/guidelines.xhtml

REFERENCES

[1] WHO. (2013). Millions of People in the world have hearing loss that can be treated or prevented. *World Health Organization*, 20.

[2] Wilson, B. S., Tucci, D. L., Merson, M. H., & O'Donoghue, G. M. (2017). Global hearing health care: new findings and perspectives. *The Lancet*, *390*(10111), 2503–2515. https://doi.org/10.1016/S0140-6736(17)31073-5

[3] Stevens, G., Group, on behalf of the G. B. of D. H. L. E., Flaxman, S., Group, on behalf of the G. B. of D. H. L. E., Brunskill, E., Group, on behalf of the G. B. of D. H. L. E., … Group, on behalf of the G. B. of D. H. L. E. (2013). Global and regional hearing impairment prevalence: an analysis of 42 studies in 29 countries. *European Journal of Public Health*, *23*(1), 146–152. Retrieved from http://dx.doi.org/10.1093/eurpub/ckr176

[4] Lutman, M. E., & Spencer, H. S. (1990). Occupational noise and demographic factors in hearing. *Acta Oto-Laryngologica. Supplementum*, *476*, 74–84.

[5] Tsimpida, D., Kaitelidou, D., & Galanis, P. (2018). Determinants of Health-related Quality of Life (HRQoL) among Deaf and Hard of Hearing Adults in Greece: a Cross-Sectional Study. *Archives of Public Health.*, 76(55)

[6] Scholes, S., Biddulph, J., Davis, A., & Mindell, J. S. (2018). Socioeconomic differences in hearing among middle-aged and older adults: cross-sectional analyses using the Health Survey for England. *BMJ Open*, *8*(2), e019615. https://doi.org/10.1136/bmjopen-2017-019615

[7] Kawachi, I., Subramanian, S. V., & Almeida-Filho, N. (2002). A glossary for health inequalities. *Journal of Epidemiology & Community Health*, *56*(9), 647-652.

[8] Marmot, M. G., Allen, J., Goldblatt, P., Boyce, T., McNeish, D., Grady, M., & Geddes, I. (2010). Fair society, healthy lives: Strategic review of health inequalities in England post-2010.

[9] Gopinath, B., Flood, V. M., McMahon, C. M., Burlutsky, G., Smith, W., & Mitchell, P. (2010). The effects of smoking and alcohol consumption on age-related hearing loss: the Blue Mountains Hearing Study. *Ear and hearing*, *31*(2), 277-282.

[10] Zhan, W., Cruickshanks, K. J., Klein, B. E. K., Klein, R., Huang, G.-H., Pankow, J. S., … Tweed, T. S. (2011). Modifiable determinants of hearing impairment in adults. *Preventive Medicine*, *53*(4–5), 338–342. https://doi.org/10.1016/j.ypmed.2011.08.012

[11] Curhan, S. G., Eavey, R., Wang, M., Stampfer, M. J., & Curhan, G. C. (2013). Body mass index, waist circumference, physical activity, and risk of hearing loss in women. *The American journal of medicine*, *126*(12), 1142-e1.

[12] Galobardes, B., Shaw, M., Lawlor, D. A., Lynch, J. W., & Smith, G. D. (2006). Indicators of socioeconomic position (part 1). *J Epidemiol Community Health*, *60*. https://doi.org/10.1136/jech.2004.023531

[13] Health Profile for England. (2018).
Retrieved from https://www.gov.uk/government/publications/health-profile-for-england-2018

[14] Poortinga, W. (2007). The prevalence and clustering of four major lifestyle risk factors in an English adult population. *Preventive Medicine*, *44*(2), 124–128. https://doi.org/10.1016/j.ypmed.2006.10.006

[15] Steptoe, A., Breeze, E., Banks, J., & Nazroo, J. (2012). Cohort profile: the English longitudinal study of ageing. *International journal of epidemiology*, *42*(6), 1640-1648.

For peer review only - http://bmjopen.bmj.com/site/about/guidelines.xhtml

[16] Banks, J., Blake, M., Clemens, S., Marmot, M., Nazroo, J., Oldfield, Z., Oskala, A., Phelps, A., Rogers, N., Steptoe, A. (2018). English Longitudinal Study of Ageing: Waves 0-8, 1998-2017. [data collection]. 29th Edition. UK Data Service. SN: 5050, http://doi.org/10.5255/UKDA-SN-5050-16

[17] Siemens Audiologische Technik GmbH. Hear Check Screener User Guide. PUBLICIS; 2007 [on-line]. Available at http://www.connevans.info/image/connevans/38shearcheck.pdf

[18] Fellizar-Lopez, K. R., Abes, G. T., Reyes-Quintos, M., Rina, T., Tantoco, M., & Leah, S. (2011). Accuracy of Siemens HearCheck™ Navigator as a Screening Tool for Hearing Loss. *Philippine Journal of Otolaryngology Head and Neck Surgery*, *26*(1), 10-15.

[19] Davis, A., Smith, P., Ferguson, M., Stephens, D., & Gianopoulos, I. (2007). Acceptability, benefits and costs of early screening for hearing disability study tests and models.pdf. *Health Technology Assessment*, *11*(42). https://doi.org/10.3310/hta11420

[20] Benova, L., Grundy, E., & Ploubidis, G. B. (2014). Socioeconomic position and health-seeking behavior for hearing loss among older adults in England. *Journals of Gerontology Series B: Psychological Sciences and Social Sciences*, *70*(3), 443-452.

[21] Bjorntorp, P., Bray, G. A., Carroll, K. K., Chuchalin, A., Dietz, W. H., Ehrlich, G. E., … Zimmet, P. (2000). Obesity : Preventing and Managing the Global Epidemic. *WHO Technical Report Series*. https://doi.org/ISBN 92 4 120894 5

[22] Department of Health. (2016). UK Chief Medical Officers' Low Risk Drinking Guidelines, (August), 11. Retrieved from https://assets.publishing.service.gov.uk/government/uploads/system/uploads/attachment_data/file/545937/UK_CMOs__report.pdf

[23] StataCorp (2015). Stata Statistical Software: Release 14. College Station, TX: StataCorp LP.

[24] Andrade, F. C. D., & Lopez-Ortega, M. (2017). Educational Differences in Health Among Middle-Aged and Older Adults in Brazil and Mexico. *Journal of Aging and Health*, 898264317705781. https://doi.org/10.1177/0898264317705781

[25] Sung, J., Sim, C., Lee, C.-R., Yoo, C.-I., Lee, H., Kim, Y., & Lee, J. (2013). Relationship of cigarette smoking and hearing loss in workers exposed to occupational noise. *Annals of Occupational and Environmental Medicine*, *25*(1), 8. https://doi.org/10.1186/2052-4374-25-8

[26] Martin, L. G., Schoeni, R. F., Andreski, P. M., & Jagger, C. (2012). Trends and inequalities in late-life health and functioning in England. *Journal of Epidemiology and Community Health*, *66*(10), 874–880. https://doi.org/10.1136/jech-2011-200251

[27] Pierre, P. V., Fridberger, A., Wikman, A., & Alexanderson, K. (2012). Self-reported hearing difficulties, main income sources, and socio-economic status; a cross-sectional population-based study in Sweden. *BMC Public Health*, *12*, 874. https://doi.org/10.1186/1471-2458-12-874

[28] Rosenhall, U., Jonsson, R., & Soderlind, O. (1999). Self-assessed hearing problems in Sweden: a demographic study. *Audiology : Official Organ of the International Society of Audiology*, *38*(6), 328–334.

[29] Davis, A. C. (1989). The prevalence of hearing impairment and reported hearing disability among adults in Great Britain. *International Journal of Epidemiology*, *18*(4), 911–917.

[30] Cruickshanks, K. J., Wiley, T. L., Tweed, T. S., Klein, B. E. K., Klein, R., Mares-Perlman, J. A., & Nondahl, D. M. (1998a). Prevalence of Hearing Loss in Older Adults in Beaver Dam,

For peer review only - http://bmjopen.bmj.com/site/about/guidelines.xhtml

Wisconsin: The Epidemiology of Hearing Loss Study. *American Journal of Epidemiology*. https://doi.org/10.1093/oxfordjournals.aje.a009713

[31] Mamo, S. K., Nieman, C. L., & Lin, F. R. (2016). Prevalence of Untreated Hearing Loss by Income among Older Adults in the United States. *Journal of Health Care for the Poor and Underserved*, *27*(4), 1812–1818. https://doi.org/10.1353/hpu.2016.0164

[32] Tsimpida, D., Kaitelidou, D., & Galanis, P. (2018). Barriers to the Use of Health Services among Deaf and Hard of Hearing Adults in Greece: a Cross-Sectional Study. *European Journal for Person Centred Healthcare*, 6(4) http://dx.doi.org/10.5750/ejpch.v6i4.1566

[33] Üçler, R., Turan, M., Garça, F., Acar, İ., Atmaca, M., & Çankaya, H. (2016). The association of obesity with hearing thresholds in women aged 18–40 years. *Endocrine*, *52*(1), 46–53. https://doi.org/10.1007/s12020-015-0755-y

[34] Bishop, C. (2012). The Ear is a Window to the Heart: A Modest Argument for a Closer Integration of Medical Disciplines. *Otolaryngology*, *02*(04), 4172. https://doi.org/10.4172/2161-119X.1000e108

[35] Hoffman, H. J., Dobie, R. A., Losonczy, K. G., Themann, C. L., & Flamme, G. A. (2017). Declining prevalence of hearing loss in US adults aged 20 to 69 years. *JAMA otolaryngology–head & neck surgery*, *143*(3), 274-285.

[36] Cruickshanks, K. J., Dhar, S., Dinces, E., Fifer, R. C., Gonzalez, F., Heiss, G., ... & Torre, P. (2015). Hearing impairment prevalence and associated risk factors in the Hispanic Community Health Study/Study of Latinos. *JAMA Otolaryngology–Head & Neck Surgery*, *141*(7), 641-648.

[37] Lin, F. R., Thorpe, R., Gordon-Salant, S., & Ferrucci, L. (2011). Hearing loss prevalence and risk factors among older adults in the United States. *Journals of Gerontology Series A: Biomedical Sciences and Medical Sciences*, *66*(5), 582-590.

[38] WHO (2015). Fact sheets: non-communicable diseases Retrieved from https://www.who.int/topics/noncommunicable_diseases/factsheets/en/

[39] Gochman, D. S. (Ed.). (2013). *Handbook of health behavior research II: provider determinants*. Springer Science & Business Media.

For peer review only - http://bmjopen.bmj.com/site/about/guidelines.xhtml

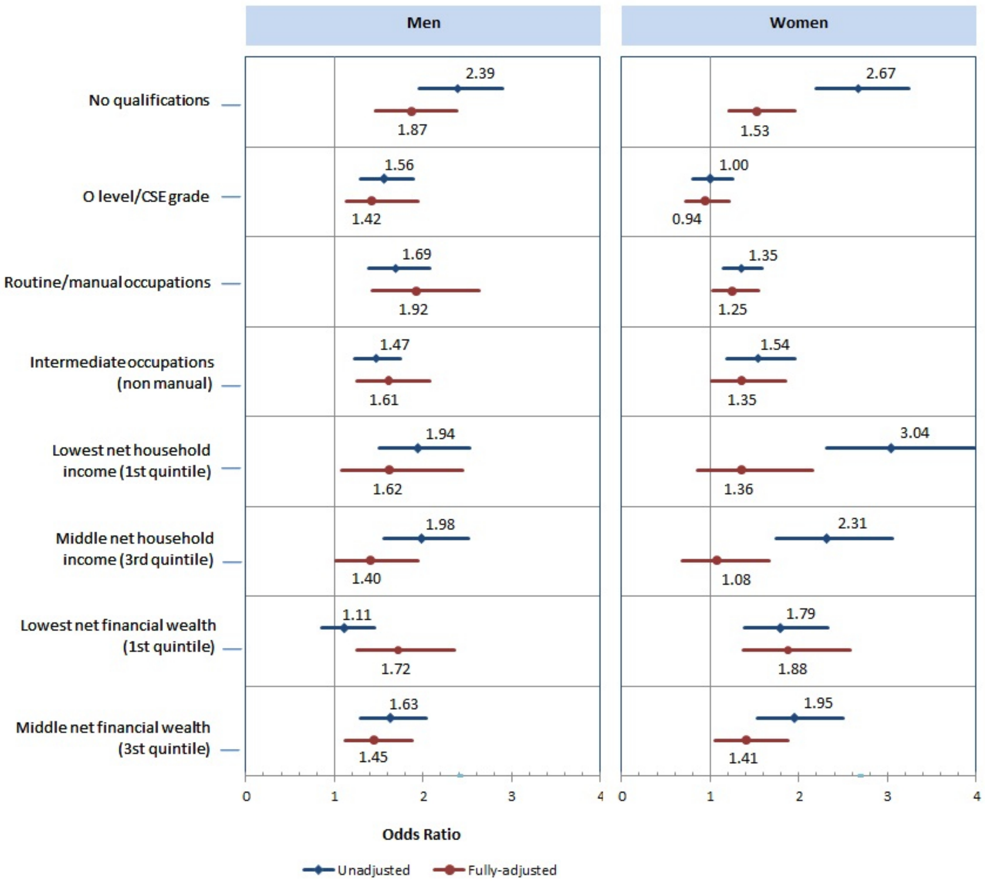

Figure 1.
Associations between socioeconomic position and hearing loss in middle aged and older adults (N=8,529, aged 50-89). Indicators of SEP were categories of the highest educational attainment (degree/higher education as a reference), tertiles of self-reported occupation based on the National Statistics Socio-economic Classification (NS-SEC) (managerial and professional as reference), quintiles of the net household income (first quintile lowest; fifth quintile highest) and quintiles of the total non-pension wealth that is reported at the household level (first quintile lowest; fifth quintile highest). Lines represent OR (outcome=hearing loss) and its 95% CI. Model A (rhombus): unadjusted. Model B (circles): adjusted for age, marital status, retirement status, body mass index, tobacco consumption, alcohol consumption and physical activity.

72x67mm (300 x 300 DPI)

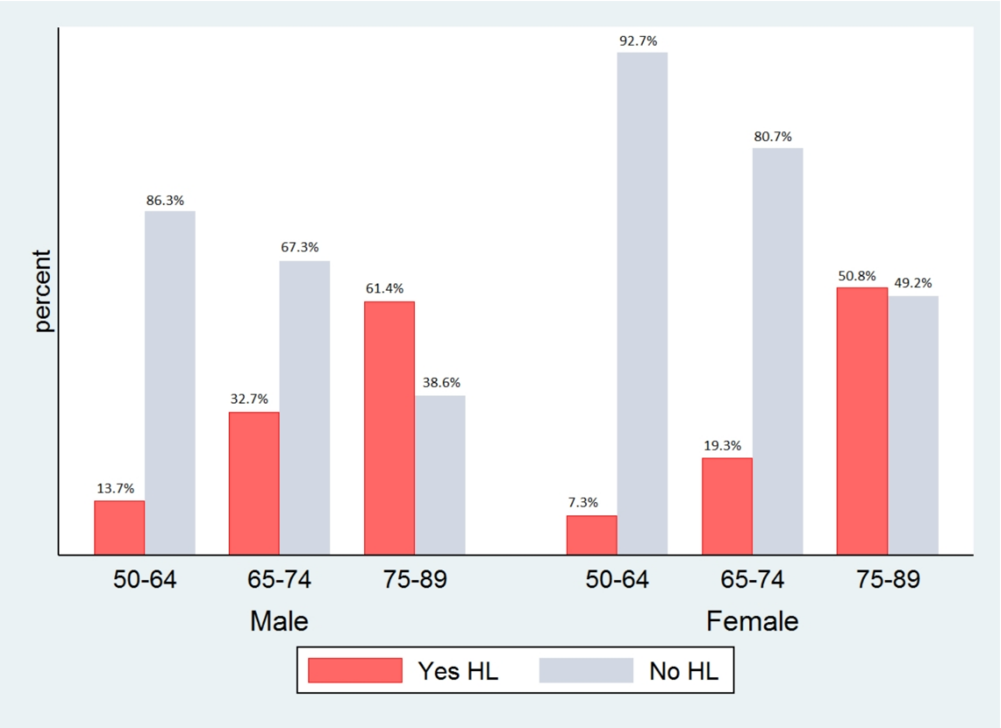

Figure 2.
Hearing loss by age group and gender* (N=8,529 participants, aged 50-89, from the seventh wave of the English Longitudinal Study of Ageing (ELSA).
Hearing loss was defined as >35 dB HL at 3.0 kHz, in the better-hearing ear.
*Prevalence estimates for males (N=3,728) and females (N=4,801).

STROBE Statement—Checklist of items that should be included in reports of ***cross-sectional studies***

	Item No	Recommendation
**Title and abstract**	1	(*a*) Indicate the study's design with a commonly used term in the title or the abstract **(Page 1)**
		(*b*) Provide in the abstract an informative and balanced summary of what was done and what was found **(Page 2)**
**Introduction**		
Background/rationale	2	Explain the scientific background and rationale for the investigation being reported **(Page 4)**
Objectives	3	State specific objectives, including any prespecified hypotheses **(Pages 4-5)**
**Methods**		
Study design	4	Present key elements of study design early in the paper **(Page 5)**
Setting	5	Describe the setting, locations, and relevant dates, including periods of recruitment, exposure, follow-up, and data collection **(Page 5)**
Participants	6	(*a*) Give the eligibility criteria, and the sources and methods of selection of participants **(Page 5)**
Variables	7	Clearly define all outcomes, exposures, predictors, potential confounders, and effect modifiers. Give diagnostic criteria, if applicable **(Pages 6-9)**
Data sources/ measurement	8*	For each variable of interest, give sources of data and details of methods of assessment (measurement). Describe comparability of assessment methods if there is more than one group **(Page 6)**
Bias	9	Describe any efforts to address potential sources of bias **(Page 6)**
Study size	10	Explain how the study size was arrived at **(Page 5)**
Quantitative variables	11	Explain how quantitative variables were handled in the analyses. If applicable, describe which groupings were chosen and why **(Page 7)**
Statistical methods	12	(*a*) Describe all statistical methods, including those used to control for confounding **(Page 9)**
		(*b*) Describe any methods used to examine subgroups and interactions **(Page 9)**
		(*c*) Explain how missing data were addressed **(Page 9)**
		(*d*) If applicable, describe analytical methods taking account of sampling strategy **(Page 9)**
		(*e*) Describe any sensitivity analyses **(Pages 9-10)**

Results

For peer review only - http://bmjopen.bmj.com/site/about/guidelines.xhtml

Participants	13*	(a) Report numbers of individuals at each stage of study—eg numbers potentially eligible, examined for eligibility, confirmed eligible, included in the study, completing follow-up, and analysed **(Page 9)**
		(b) Give reasons for non-participation at each stage **(Page 9)**
Descriptive data	14*	(a) Give characteristics of study participants (eg demographic, clinical, social) and information on exposures and potential confounders **(Page 5)**
		(b) Indicate number of participants with missing data for each variable of interest **(Page 9)**
Outcome data	15*	Report numbers of outcome events or summary measures **(Page 10)**
Main results	16	(*a*) Give unadjusted estimates and, if applicable, confounder-adjusted estimates and their precision (eg, 95% confidence interval). Make clear which confounders were adjusted for and why they were included **(Page 11)**
		(*b*) Report category boundaries when continuous variables were categorized (not relevant)
		(*c*) If relevant, consider translating estimates of relative risk into absolute risk for a meaningful time period (not relevant)
Other analyses	17	Report other analyses done—eg analyses of subgroups and interactions, and sensitivity analyses **(Page 12)**
**Discussion**		
Key results	18	Summarise key results with reference to study objectives **(Page 12)**
Limitations	19	Discuss limitations of the study, taking into account sources of potential bias or imprecision. Discuss both direction and magnitude of any potential bias **(Page 13)**
Interpretation	20	Give a cautious overall interpretation of results considering objectives, limitations, multiplicity of analyses, results from similar studies, and other relevant evidence **(Page 15)**
Generalisability	21	Discuss the generalisability (external validity) of the study results **(Page 13)**
**Other information**		
Funding	22	Give the source of funding and the role of the funders for the present study and, if applicable, for the original study on which the present article is based **(Page 17)**

*Give information separately for exposed and unexposed groups.

Note: An Explanation and Elaboration article discusses each checklist item and gives methodological background and published examples of transparent reporting. The STROBE checklist is best used in conjunction with this article (freely available on the Web sites of PLoS Medicine at http://www.plosmedicine.org/, Annals of Internal Medicine at

For peer review only - http://bmjopen.bmj.com/site/about/guidelines.xhtml

http://www.annals.org/, and Epidemiology at http://www.epidem.com/). Information on the STROBE Initiative is available at www.strobe-statement.org.

For peer review only

For peer review only - http://bmjopen.bmj.com/site/about/guidelines.xhtml
